# Computed tomography and magnetic resonance imaging features of adnexal and leiomyoma torsion: correlation with laparoscopic findings

**DOI:** 10.1007/s11604-025-01881-8

**Published:** 2025-11-10

**Authors:** Hideyuki Fukui, Takahiro Tsuboyama, Hiromitsu Onishi, Takashi Ota, Atsushi Nakamoto, Toru Honda, Kengo Kiso, Shohei Matsumoto, Koki Kaketaka, Takumi Tanigaki, Masatoshi Hori, Mitsuaki Tatsumi, Noriyuki Tomiyama

**Affiliations:** 1 Department of Diagnostic and Interventional Radiology, Osaka University Graduate School of Medicine, D1, 2-2, Yamadaoka, Suita, Osaka 565-0871 Japan; 2 Department of Radiology, Kobe University Graduate School of Medicine, Kobe, Japan; 3 Department of Medical Physics and Engineering, Osaka University Graduate School of Medicine, Suita, Osaka Japan; 4 Department of Artificial Intelligence Diagnostic Radiology, Osaka University Graduate School of Medicine, Suita, Osaka Japan

**Keywords:** Adnexal torsion, Leiomyoma torsion, Magnetic resonance imaging, Computed tomography, Laparoscopy

## Abstract

Adnexal torsion and pedunculated subserosal leiomyoma torsion are significant gynecological emergencies requiring prompt recognition and surgical intervention. Although ultrasound remains the primary imaging modality, cross-sectional imaging with computed tomography and magnetic resonance imaging plays a crucial role in diagnosis, particularly in complex cases. This review provides a comprehensive analysis of the imaging features of various types of torsion, with direct correlation with laparoscopic findings. We describe key imaging features across different modalities, focusing on specific manifestations of ovarian torsion variants, including massive ovarian edema, mature cystic teratoma, fibroma, and mucinous cystadenoma. Special attention was given to isolated fallopian tube torsion and its subtypes and the unique features of leiomyoma torsion. Understanding these imaging features and their correlation with laparoscopic findings is crucial for accurate diagnosis and appropriate surgical planning. This review emphasizes the importance of recognizing specific imaging patterns that can help guide clinical decision-making and improve patient outcomes through timely intervention.

## Introduction

Torsion of adnexal structures and pedunculated subserosal leiomyomas represent significant gynecological emergencies requiring prompt recognition and intervention. Adnexal torsion, being the fifth most common gynecological surgical emergency, accounts for approximately 2.7% of acute gynecological conditions requiring surgery [1, 2]. Similarly, although less common, torsion of pedunculated subserosal leiomyomas can present with acute symptoms requiring immediate surgical attention [3–5].

The diagnostic challenge of these conditions lies in their variable and often nonspecific clinical presentations, which can range from acute to intermittent symptoms [6–8]. This diagnostic uncertainty frequently leads to delays in appropriate intervention, potentially resulting in tissue necrosis and loss of organ function [6, 7, 9, 10]. The integration of imaging findings with laparoscopic observations significantly enhanced our understanding of the pathophysiology and various presentations of these conditions [4, 11–13].

Recent advances in both imaging technology and minimally invasive surgical techniques have revolutionized the approach to diagnosis and treatment. Laparoscopy not only is the gold standard for diagnosis but also provides crucial insights into the mechanical aspects and progression of torsion that complement imaging findings [10, 14–16]. Although ultrasound remains the first-line imaging modality, cross-sectional imaging with computed tomography (CT) and magnetic resonance imaging (MRI) offers additional valuable information, particularly in complex cases [16–18].

This review aims to provide a comprehensive analysis of adnexal and leiomyoma torsion, correlating imaging features with laparoscopic findings. This study focused on key diagnostic features across different imaging modalities, potential pitfalls, and the crucial role of laparoscopic correlation in understanding these conditions. Special emphasis was placed on how imaging findings can guide surgical planning and decision-making.

## Normal anatomy and pathophysiology

### Normal anatomy of the female pelvic structures and supporting ligaments

**Fig. 1:**
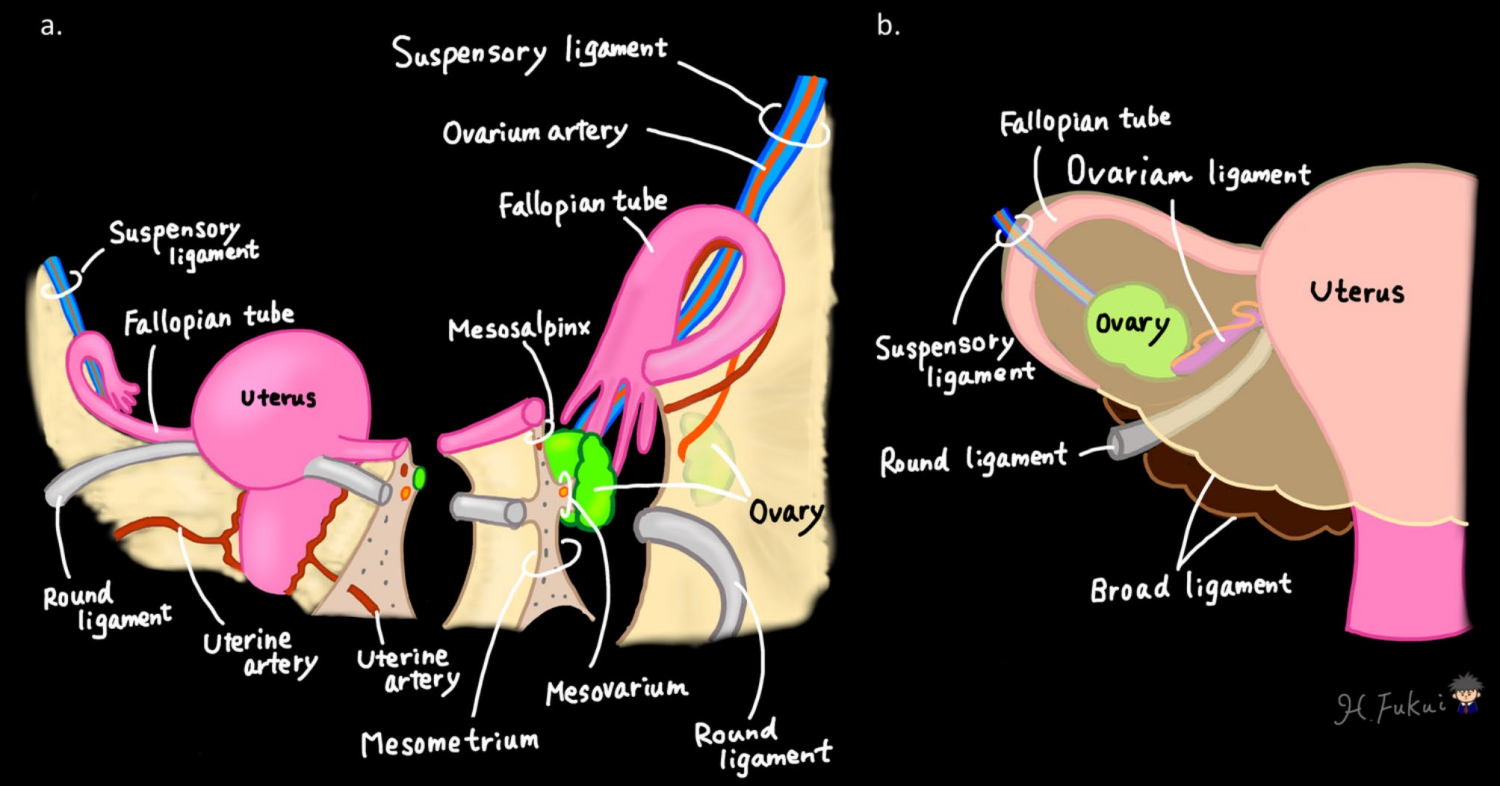
Normal anatomical illustration with the key structures labeled. **a** Lateral view showing the uterus, fallopian tube, and ovary with their supporting ligaments. **b** Coronal view demonstrating the relationship between the uterus, ovary, and supporting structures. The key structures depicted include the uterus, ovaries, fallopian tubes, and various ligaments that support these organs within the pelvic cavity. These anatomical relationships are crucial for understanding the mechanisms underlying adnexal and leiomyoma torsion

A thorough understanding of the normal pelvic anatomy and supporting ligaments is essential for recognizing the pathological changes that occur in adnexal and leiomyoma torsion [15, 19–21]. The ovaries are paired organs typically located in the lateral pelvic wall at the level of the pelvic brim, anterior to the sacroiliac joints [8, 14, 15, 22]. Each ovary is suspended by several key ligamentous structures that play crucial roles in both normal positioning and the development of torsion (Fig. 1).

The suspensory ligament (infundibulopelvic ligament) extends from the upper pole of the ovary to the lateral pelvic wall and contains the ovarian vessels and nerves [15, 20–22]. The ligament serves as the primary axis on which torsion can occur. The ovarian ligament (utero-ovarian ligament) connects the ovary to the lateral aspect of the uterus and contains branches of uterine vessels that provide secondary blood supply to the ovary [15, 21, 22].

The broad ligament is a double-layered peritoneal fold that extends from the lateral pelvic wall to the uterus and encompasses several important structures. The superior portion contains the fallopian tube within its free edge (mesosalpinx), and the lateral portion contains the ovary (mesovarium) [14, 15, 22]. This anatomical arrangement creates potential spaces in which fluid can accumulate and masses can develop, potentially predisposing to torsion [15, 21].

The fallopian tubes, which extend laterally from the uterine cornua, are divided into four segments: the intramural, isthmic, ampullary, and infundibular portions [23]. The tube’s relatively mobile nature, particularly its fimbriated end, combined with its rich vascular supply, makes it susceptible to torsion, either in isolation or in conjunction with the ovary [23].

**Fig. 2:**
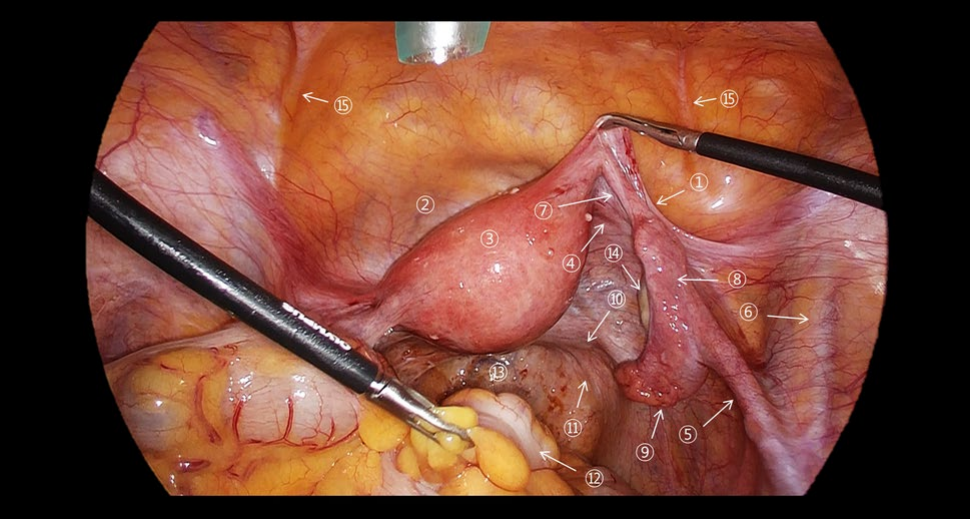
Normal laparoscopic anatomy of the female pelvis demonstrating the key anatomical structures and their relationships. Numbers indicate ① round ligament of the uterus, ② vesicouterine pouch, ③ uterus, ④ ovarian ligament, ⑤ suspensory ligament of the ovary, ⑥ right external iliac vein, ⑦ isthmus of the uterine tube, ⑧ ampulla of the uterine tube, ⑨ infundibulum of the uterine tube, ⑩ uterosacral ligament, ⑪ right ureter, ⑫ rectum, ⑬ Pouch of Douglas, ⑭ right ovary, and ⑮ medial umbilical fold

**Fig. 3:**
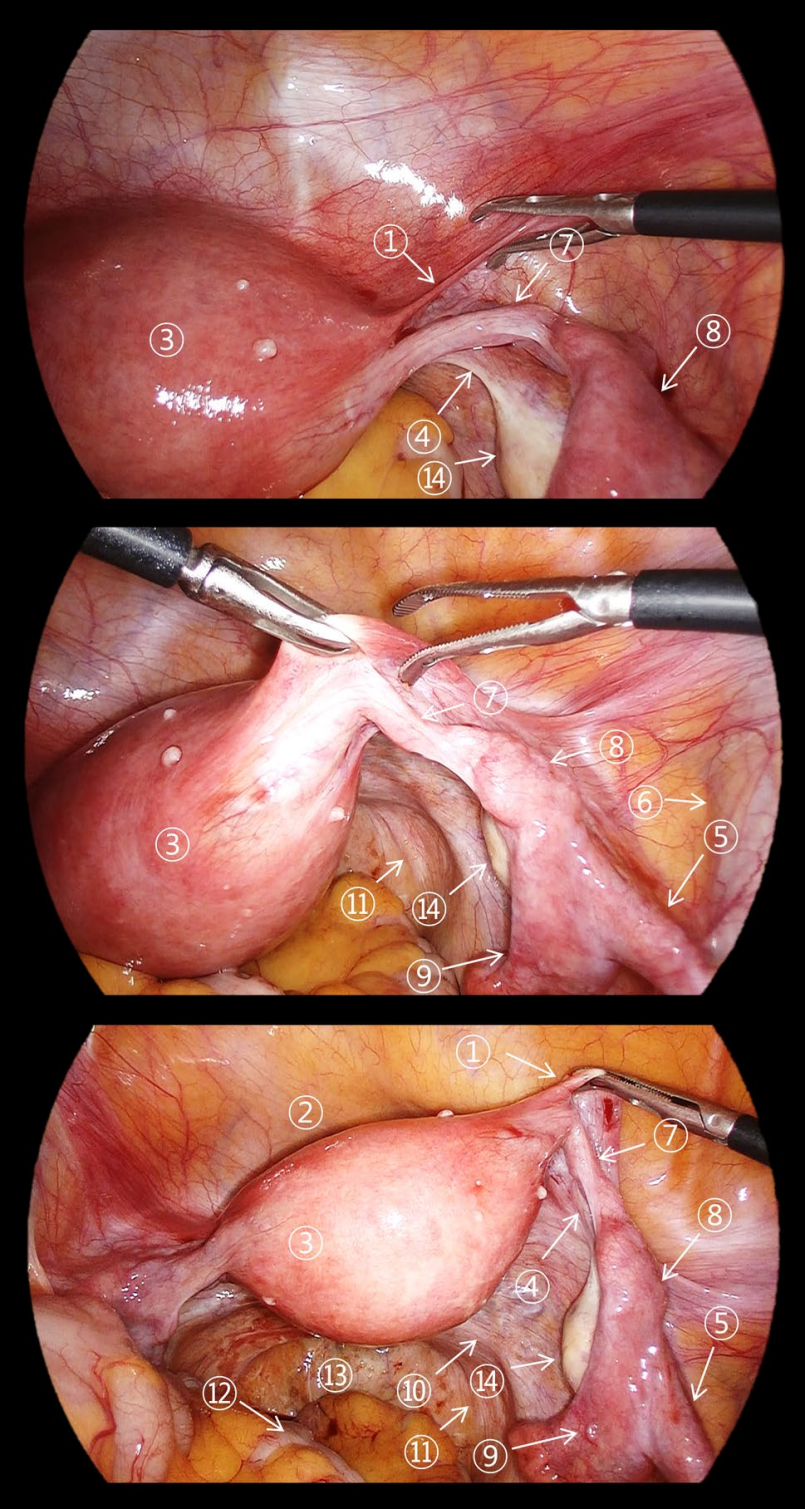
Normal laparoscopic anatomy of the female pelvis demonstrating the key anatomical structures and their relationships. Numbers indicate ① round ligament of the uterus, ② vesicouterine pouch, ③ uterus, ④ ovarian ligament, ⑤ suspensory ligament of the ovary, ⑥ right external iliac vein, ⑦ isthmus of the uterine tube, ⑧ ampulla of the uterine tube, ⑨ infundibulum of the uterine tube, ⑩ uterosacral ligament, ⑪ right ureter, ⑫ rectum, ⑬ Pouch of Douglas, and ⑭ right ovary

Understanding this complex anatomical network is crucial for interpreting both the imaging findings and laparoscopic appearances of adnexal and leiomyoma torsion. Recognition of normal anatomical relationships helps identify subtle changes that occur in early torsion and more dramatic changes seen in advanced cases [12, 14, 24]. Figures 2 and 3 present the normal laparoscopic anatomy of the female pelvis, highlighting the key anatomical structures and their relationships. These images provide a foundation for understanding pathological changes during torsion.

### Vascular supply and its importance in torsion

The ovary has a unique dual blood supply system that significantly influences the progression and imaging appearance of torsion [14, 25]. Blood supply primarily originates from the ovarian arteries, which arise directly from the abdominal aorta below the renal arteries and travel within the suspensory ligament. The secondary supply is derived from the ovarian branches of the uterine arteries, which course through the utero-ovarian ligament and anastomose with the ovarian arteries [22, 25].

This dual vascular arrangement has important implications for the pathophysiology of torsion. When torsion occurs, the initial vascular compromise typically affects venous and lymphatic drainage before arterial flow because of the different vessel wall characteristics [15, 25, 26].

The progression of vascular compromise during torsion follows a predictable pattern: initial venous and lymphatic obstruction leads to ovarian edema and enlargement, which can further compress the vessels and eventually compromise arterial flow [10, 14, 15, 26]. This process can be intermittent because partial torsion can spontaneously resolve and recur. The persistence of arterial flow in venous obstruction can lead to hemorrhagic infarction, which has important implications for tissue viability [11, 15, 27].

Understanding this vascular anatomy is crucial for the interpretation of imaging findings. The “whirlpool sign,” representing the twisted vascular pedicle, is a key diagnostic feature that is visible across multiple imaging modalities [14, 15, 26, 28]. Furthermore, the pattern of contrast enhancement on CT and MRI reflects the stage of vascular compromise, with early torsion showing preserved enhancement and late stages demonstrating lack of enhancement [14, 18, 20, 26].

### Mechanisms of adnexal and leiomyoma torsion

Adnexal and leiomyoma torsion share some common mechanical principles but have distinct pathophysiological mechanisms. Understanding these mechanisms is crucial for accurate diagnosis and appropriate management [3, 4, 21].

**Fig. 4:**
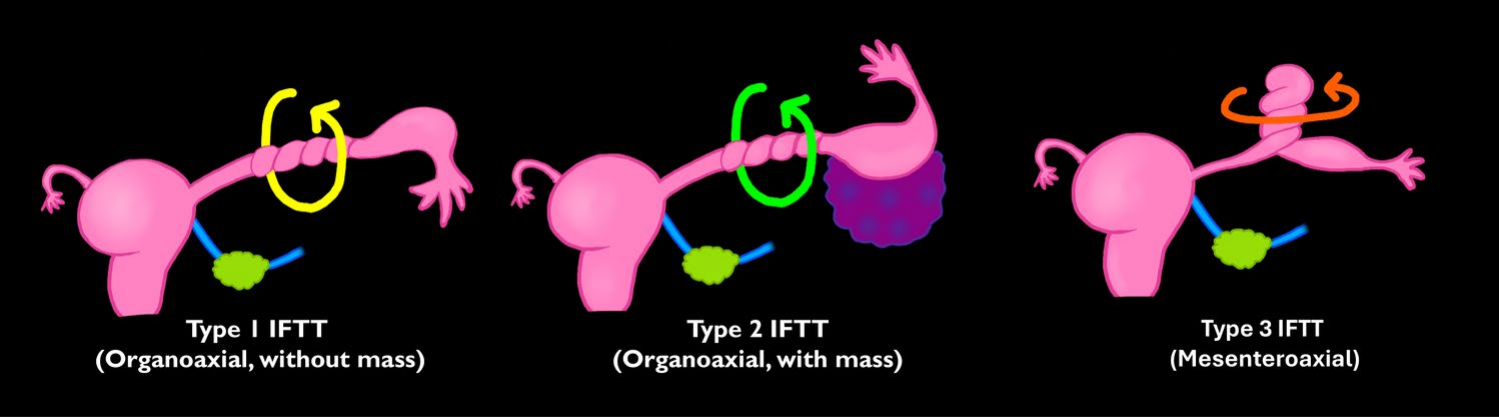
Schematic of isolated fallopian tube torsion (IFTT). Three IFTT patterns are shown: Type 1 (organoaxial form without a leading mass), Type 2 (organoaxial form with a leading mass, such as a paratubal cyst), and Type 3 (mesenteroaxial form). The tube can twist around its longitudinal axis (organoaxial) or perpendicular to its axis (mesenteroaxial)

Three main patterns of adnexal torsion can be identified: tubo-ovarian torsion, isolated ovarian torsion, and isolated fallopian tube torsion (IFTT) [1, 11, 20, 29]. The most common form is tubo-ovarian torsion, in which both the ovary and fallopian tube twist around the vascular pedicle. IFTT can be further classified into three subtypes based on the axis and presence of a leading mass: Type 1 (organoaxial form without a leading mass), Type 2 (organoaxial form with a leading mass), and Type 3 (mesenteroaxial form) (Fig. 4) [15, 30].

**Fig. 5:**
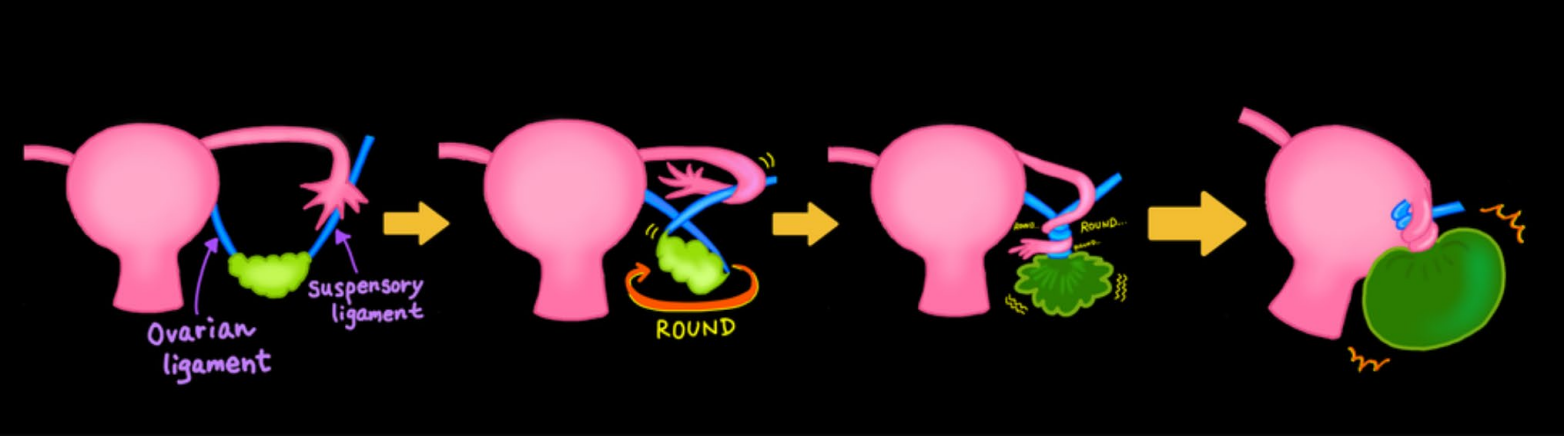
Schematic demonstrating the mechanism underlying ovarian torsion. The ovary rotates around its vascular pedicle (suspensory ligament), which contains ovarian vessels. Initial venous and lymphatic obstruction leads to ovarian enlargement and edema, which can further compromise arterial flow. The presence of an ovarian mass increases the risk of torsion by acting as a fulcrum for rotation

The presence of predisposing factors, particularly ovarian masses, significantly increases torsion risk (see detailed discussion in the “Risk Factors” section) (Fig. 5) [7, 10, 14, 17, 21, 31, 32]. The right adnexa is more commonly affected (60%–70% of cases), possibly because of the greater mobility on the right side and the stabilizing effect of the sigmoid colon on the left [8, 11, 15, 32].

**Fig. 6:**
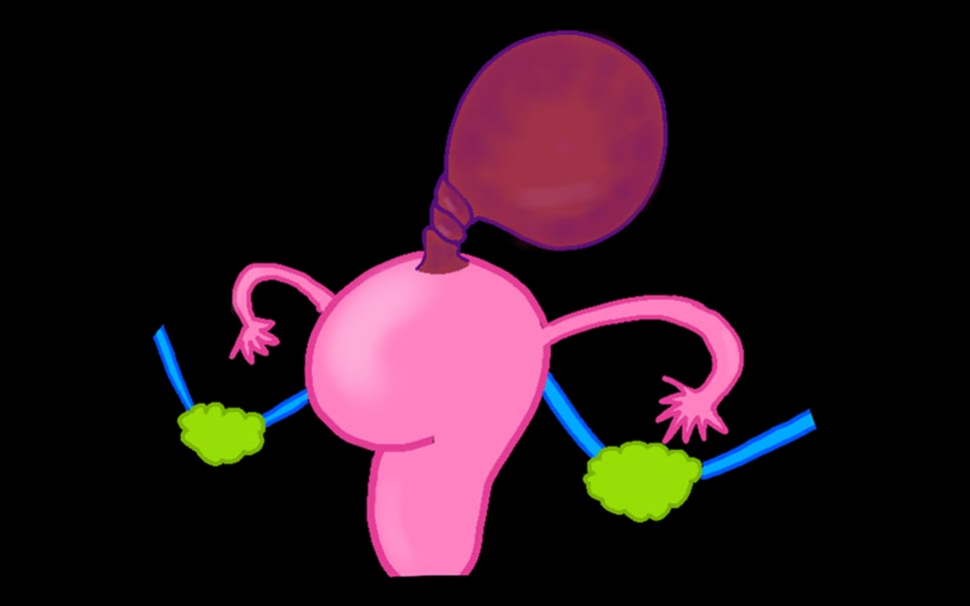
Schematic of pedunculated subserosal leiomyoma torsion. The pedunculated leiomyoma rotates around its vascular stalk, with the risk increasing proportionally with the length of the pedicle and size of the fibroid. Torsion leads to progressive vascular compromise, beginning with venous obstruction and potentially progressing to complete ischemia

Leiomyoma torsion typically occurs in pedunculated subserosal fibroids. The mechanism involves the rotation of the fibroid around its vascular pedicle, with the risk increasing proportionally with the length of the pedicle and size of the fibroid [3, 33]. Unlike adnexal torsion, leiomyoma torsion exhibits no significant laterality preference (Fig. 6).

Both conditions can present with varying degrees of rotation, from partial to complete torsion. The severity of symptoms and tissue damage is correlated with both the degree of rotation and the duration of vascular compromise [15, 24, 32]. Intermittent torsion can occur in both conditions, leading to chronic or recurrent symptoms that may complicate diagnosis [7, 12, 19].

## Clinical features and diagnostic challenges

### Clinical presentation

The clinical presentation of adnexal and leiomyoma torsion is often nonspecific, making early diagnosis challenging [10, 14, 15, 21]. Acute pelvic pain is a primary symptom observed in most cases of adnexal torsion and isolated tubal torsion, with some studies reporting its presence in > 90% of cases [6, 8, 11, 34]. The pain is typically unilateral, has a sudden onset, and may be sharp or cramping in nature. However, the intensity and character of pain can vary significantly among patients [6, 8, 9].

Nausea and vomiting are the next most common symptoms, occurring in approximately 70% of cases, with a particularly high incidence in pediatric patients [8, 21, 35]. When acute pelvic pain is accompanied by nausea or vomiting, the clinician’s suspicion of torsion should be increased [8, 15, 21, 35]. Other associated symptoms include fever and leukocytosis, which can misleadingly suggest inflammatory conditions [36–38].

The clinical course can be variable, ranging from acute to subacute. In some cases, patients may report similar episodes within the preceding month, reflecting possible intermittent torsion with spontaneous resolution [3, 12, 27]. This intermittent nature can delay diagnosis because symptoms may temporarily improve between episodes [6, 36].

In cases of leiomyoma torsion, clinical presentation may mimic that of adnexal torsion; however, the pain is often more localized to the site of a known fibroid [5, 39, 40]. The presence of a palpable abdominal mass that has recently become tender may provide additional diagnostic clues [14, 15, 32, 33].

Physical examination findings of adnexal torsion are typically nonspecific. Common findings include abdominal tenderness, which may be localized to the affected side, and a palpable adnexal mass when present. Cervical motion tenderness may also be present but is variable and nonspecific [1, 6, 10, 32].

Laboratory findings are generally nonspecific and may include leukocytosis and elevated inflammatory markers. However, these findings are neither sensitive nor specific for torsion [9, 15].

### Risk factors

Adnexal and leiomyoma torsion have distinct risk factors that can aid in diagnosis when considered alongside clinical and imaging findings [4, 10]. For adnexal torsion, the presence of an ovarian mass is the most significant predisposing factor, occurring in approximately 80% of cases [7, 10, 14, 17, 21, 32]. Adnexal torsion is most commonly associated with benign ovarian masses [9, 10], particularly mature cystic teratomas [41]. The size of these masses typically ranges from 5 to 10 cm in diameter [10, 14, 21, 31], although their dimensions can significantly vary. Notably, malignant masses are rarely associated with torsion, likely because of inflammatory adhesions that restrict mobility [8, 15, 25, 42].

Pregnancy and assisted reproductive technologies represent another important risk category. The incidence of adnexal torsion increases during pregnancy, particularly in the first trimester, with up to 20% of cases occurring in pregnant women [36]. Ovarian hyperstimulation syndrome poses a particular risk because enlarged ovaries are more susceptible to torsion [2]. Previous pelvic surgery and elongated ovarian ligaments, particularly in children and adolescents, may also predispose individuals to torsion [7, 8, 43].

The primary risk factor for leiomyoma torsion is the presence of a pedunculated subserosal fibroid, particularly those with a long thin stalk [3, 39]. The fibroid size and increased intra-abdominal pressure may also increase the risk of torsion [3, 5, 40].

### Diagnostic pitfalls

Several factors contribute to the diagnostic challenges of torsion. The most significant pitfall is overreliance on vascular Doppler findings. The presence of normal arterial flow does not exclude torsion due to the dual blood supply to the ovary and the possibility of intermittent torsion [8, 10, 31, 44]. In contrast, absent flow may be observed in other conditions or due to technical limitations [14, 25, 37].

Another common pitfall is the assumption that the absence of an ovarian mass excludes torsion, particularly in pediatric patients in whom normal ovaries may twist [45–47]. The intermittent nature of symptoms can also delay diagnosis because patients may present with recurring episodes of pain that spontaneously resolve [7, 12, 19].

In cases of leiomyoma torsion, the primary diagnostic pitfall is the tendency to attribute symptoms to simple fibroid degeneration or other causes of acute abdomen [3, 48]. The presence of a known fibroid may paradoxically delay the diagnosis of torsion when other signs are not carefully evaluated [3–5].

Laboratory findings can be misleading because inflammatory markers may be normal or only slightly elevated, even in cases of significant torsion [12, 15, 21, 38]. Furthermore, the presence of fever or elevated inflammatory markers may incorrectly suggest other diagnoses, such as pelvic inflammatory disease or appendicitis [8, 15, 21, 23].

## Imaging features

### Adnexal torsion

#### CT and MRI features of adnexal torsion

**Fig. 7:**
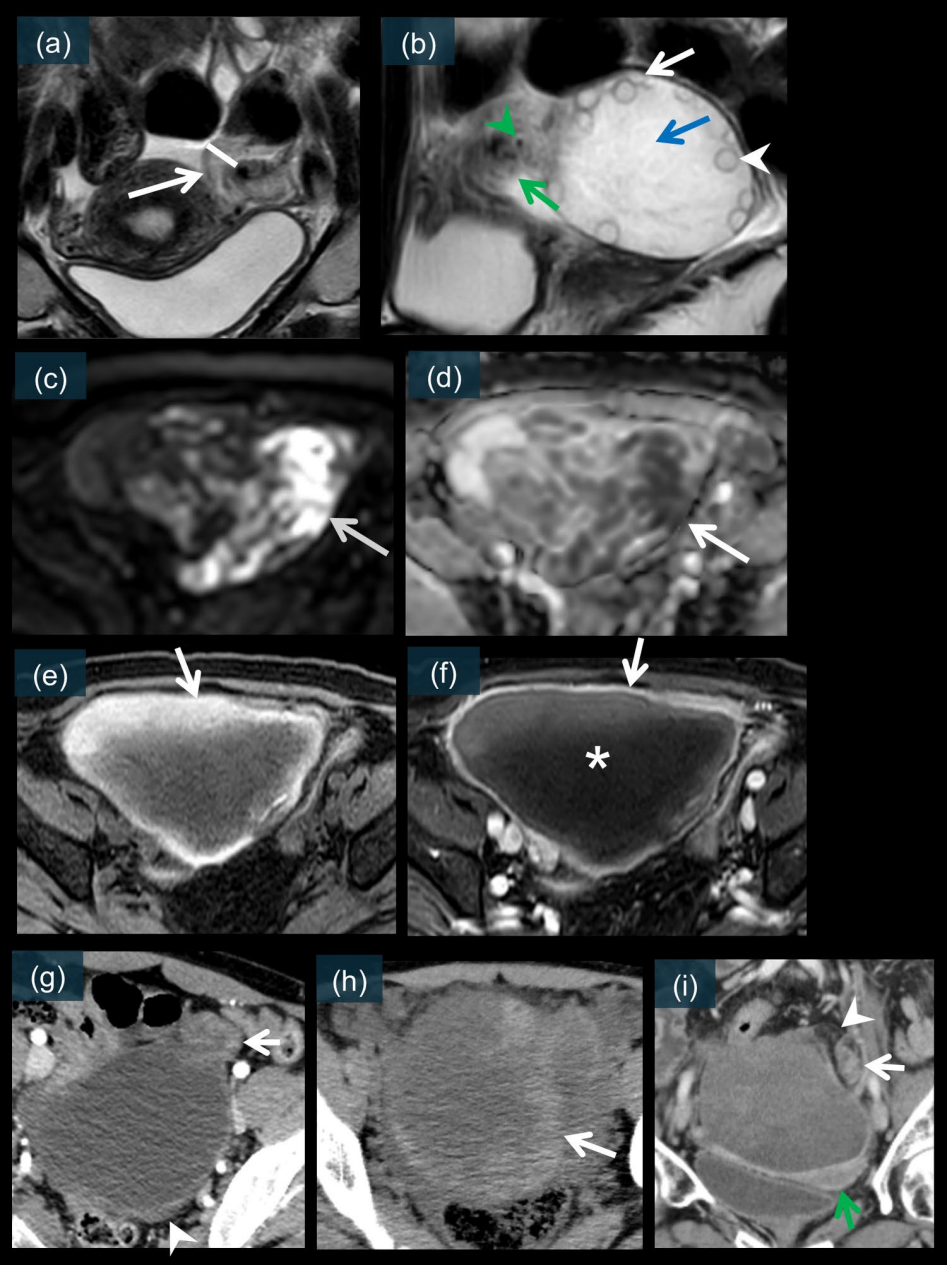
Key diagnostic signs of adnexal torsion on cross-sectional imaging. **a** Axial T2-weighted image showing fallopian tube thickening exceeding 10 mm (white arrow). **b** Sagittal T2-weighted image demonstrating the “string of pearls” sign with peripherally displaced follicles (white arrow), a perifollicular T2 hypointense rim (white arrowhead), and stromal edema (blue arrow) in massive ovarian edema. A target-like appearance of the twisted vascular pedicle is also seen (green arrow), characterized by a peripheral high T2 signal with a central flow void (green arrowhead). **c** Axial diffusion-weighted image (DWI) showing high signal intensity (white arrow) in the ovarian fibroma. **d** Apparent diffusion coefficient (ADC) map showing corresponding low values (white arrow), confirming restricted diffusion. **e** Axial T1-weighted image revealing peripheral high signal intensity (white arrow) areas indicating hemorrhage. **f** Axial contrast-enhanced T1-weighted image showing absent central enhancement (white asterisk) with preserved peripheral enhancement (white arrow). **g** Sagittal T2-weighted image demonstrating the "whirlpool sign"—a twisted vascular pedicle between the uterus and the affected adnexa (white arrow)—and stromal wall thickening (white arrowhead). **h** Unenhanced CT image showing hemorrhagic infarction with high-attenuation areas within the enlarged ovary (white arrow). **i** Contrast-enhanced CT image demonstrating uterine deviation toward the affected side (green arrow), surrounding fat stranding (white arrowhead), and a twisted pedicle (white arrow)

The most consistent feature of adnexal torsion is unilateral enlargement of the affected ovary, as shown in Figs. 7b, h, 9a, i. This enlargement occurs due to vascular congestion and edema following venous and lymphatic obstruction [12, 17, 20]. In a study involving 20 patients with surgically proven adnexal torsion, Rha et al. observed ovarian enlargement in 85% of the cases [20]. The average volume of a twisted ovary may be 28 times the size of a normal ovary [49].

**Fig. 8:**
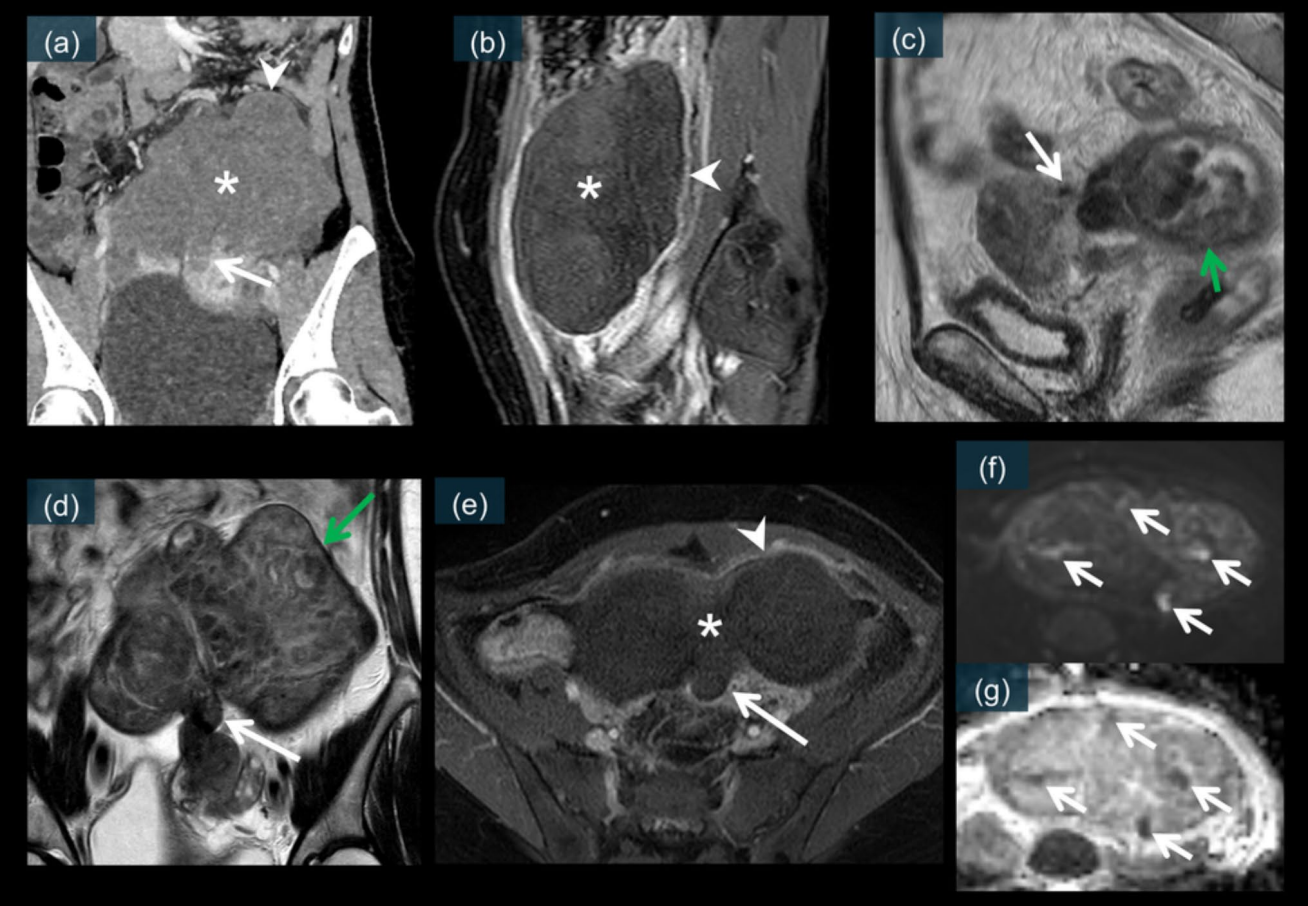
Key diagnostic signs of uterine leiomyoma torsion on cross-sectional imaging. **a** Contrast-enhanced CT showing the “dark fan sign”—decreased enhancement of the uterine myometrium adjacent to the twisted leiomyoma (white arrow)—and poor internal enhancement of the leiomyoma (white asterisk) with thin peripheral rim enhancement (white arrowhead). **b** Contrast-enhanced T1-weighted image demonstrating poor internal enhancement of the leiomyoma (white asterisk) with a thin peripheral rim enhancement (white arrowhead). **c** Sagittal T2-weighted image showing the "bridging vessel sign” (white arrow)—vascular pedicle connecting the leiomyoma to the uterus—and heterogeneous signal intensity within the leiomyoma (green arrow). **d** Coronal T2-weighted image displaying an enlarged stalk (white arrow) and heterogeneous signal intensity within the leiomyoma (green arrow). **e** Contrast-enhanced T1-weighted image showing a non-enhancing, wedge-shaped area (white arrow) corresponding to the “dark fan” sign; the mass itself shows no internal enhancement (white asterisk), with only a thin, enhanced peripheral rim (white arrowhead). **f** Diffusion-weighted image showing high signal intensity (white arrows) within the leiomyoma, indicating restricted diffusion. **g** ADC map demonstrating corresponding low values (white arrows), confirming ischemic changes

**Fig. 9:**
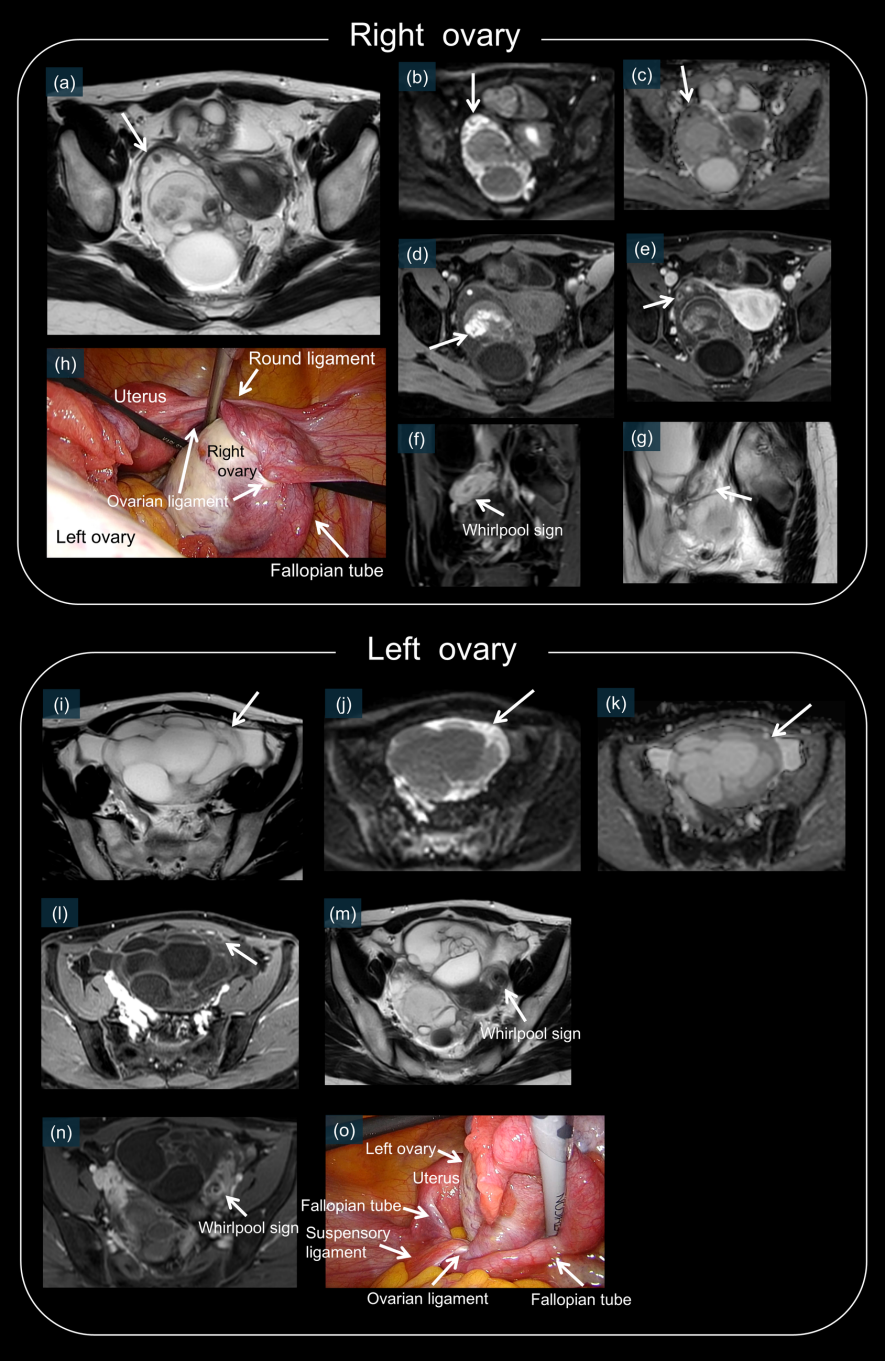
Both ovaries of a 27-year-old female patient are markedly enlarged, showing > 20 relatively large cysts, suggesting polycystic ovary syndrome. Partial hemorrhage was observed within a cyst in the right ovary on fat-suppressed T1WI (**d**, white arrow). Massive ovarian edema is partially observed in both ovaries, appearing as high signal intensity on T2WI reflecting edema (**a** and **i**, white arrows). These areas show diffusion restriction on DWI/ADC (**b**, **c**, **j**, and **k**, white arrows). Hemorrhagic infarction may be associated with a high signal intensity on DWI. On postcontrast sequences, enhancement was observed in the periphery of the ovaries and follicles (**e** and **l**, white arrows). Twisted pedicles are seen bilaterally (**f**, **g**, **m**, and **n**, white arrows), with whirlpool signs visible (**f**, **m** and **n**). **h** and **o** are laparoscopic images revealing that both ovaries are enlarged and twisted (right, 810°; left, 720°)

**Fig. 10:**
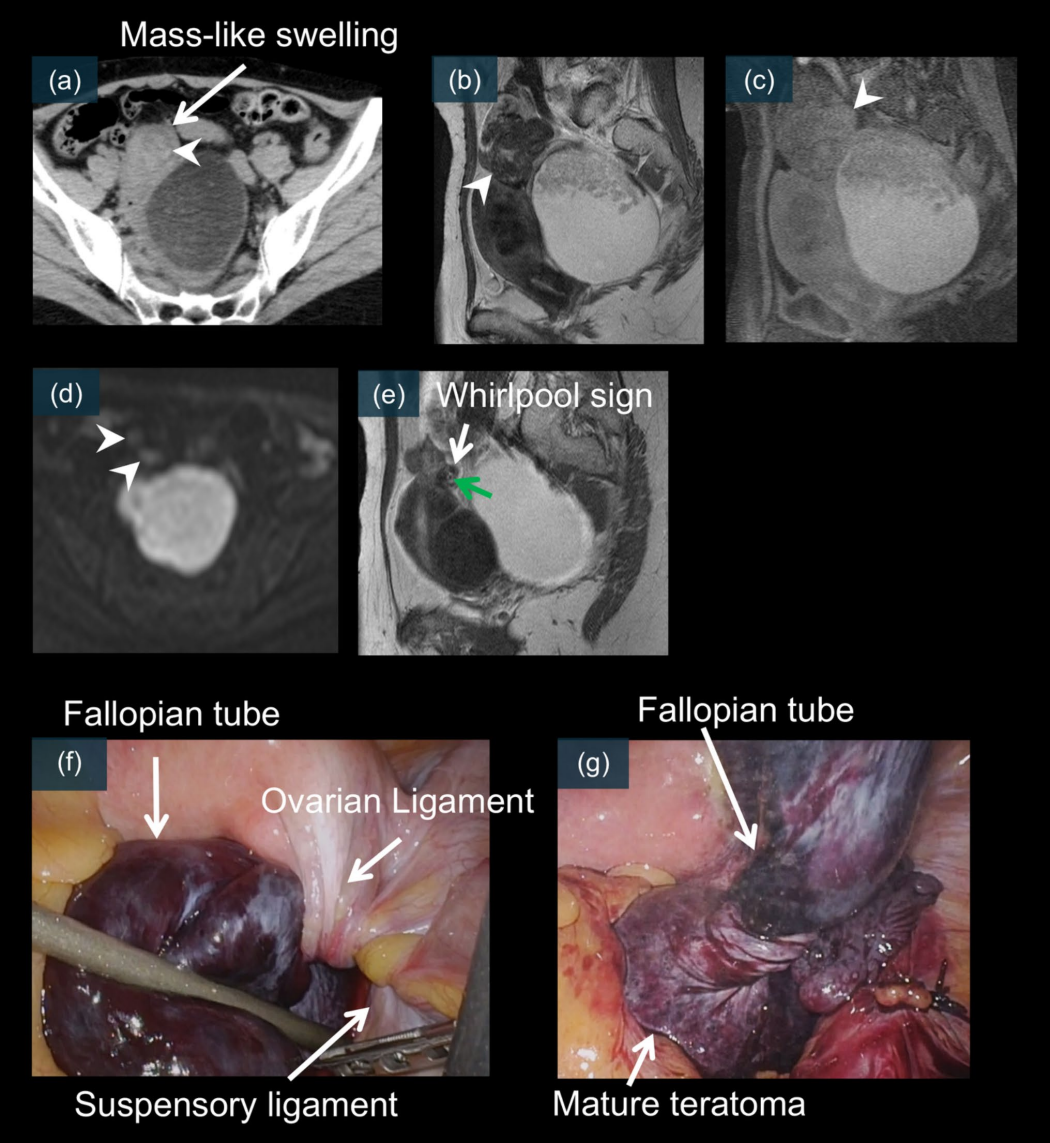
A 55-year-old female. **a** is an unenhanced CT image. Although a clear whirlpool sign is not evident, there is a mass-like structure adjacent to the mature cystic teratoma (white arrow). This is referred to as a “mass-like swelling,” indicating the presence of an enlarged fallopian tube and mesosalpinx containing dilated veins between the uterus and the twisted ovary. Heterogeneous high-density areas are visible within it (**a**, white arrowhead), which also show low signal intensity on T2WI (**b**, white arrowhead), high signal intensity on fat-suppressed T1WI (**c**, white arrowhead), and DWI (**d**, white arrowhead), likely corresponding to hemorrhage into the interstitial tissue. **e** shows a clear whirlpool sign (white arrow), with a flow void (green arrow) in the center thought to be the ovarian artery, suggesting a twisted vascular pedicle. **f** and **g** are laparoscopic images, which reveal a 720° torsion of the right mature cystic teratoma and fallopian tube

**Fig. 11:**
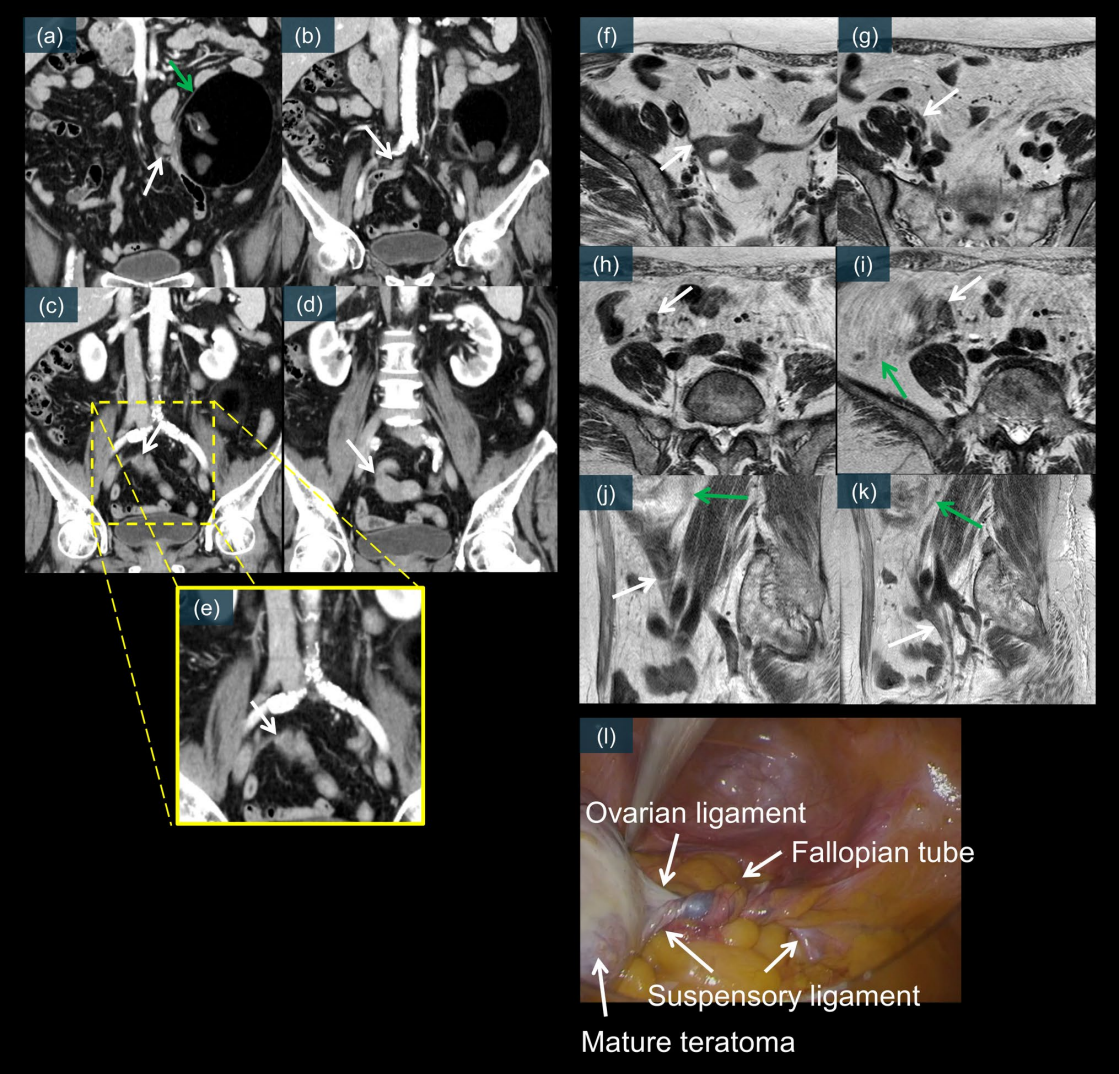
A 62-year-old female. **a–d** are contrast-enhanced CT images. The mature cystic teratoma is located in the left upper abdomen (green arrow). Although a clear whirlpool sign is not evident, a very long pedicle is observed continuing from the right uterine cornu (white arrows). A partially enhancing thin vascular structure is visible within the pedicle, suspected to be the ovarian artery (**e**, white arrow). **f–k** are axial and sagittal T2-weighted images. The mature cystic teratoma has moved to the right upper abdomen (green arrows), and the pedicle is depicted as a homogeneous low-signal-intensity structure (white arrows). No abnormal signals are observed on DWI or T1WI. **l** is a laparoscopic image, which reveals a 720° torsion of the right mature cystic teratoma and fallopian tube

**Fig. 12:**
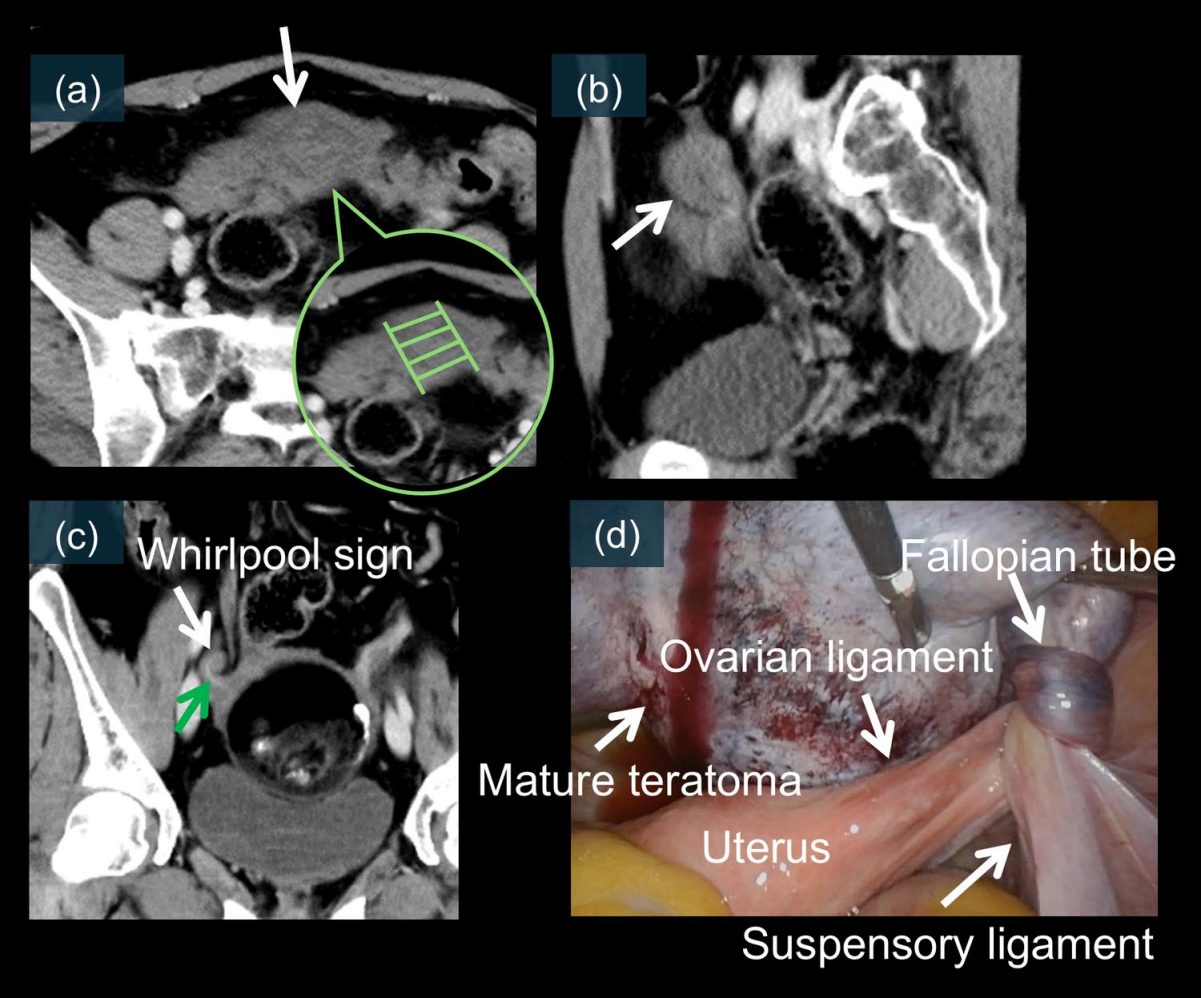
A 26-year-old female. **a–c** are axial, sagittal, and coronal contrast-enhanced CT images. The mature cystic teratoma is located in the midline of the pelvis. Although a clear whirlpool sign is not evident on the axial images, a step-ladder pattern of ovarian vessels cranial to the mature cystic teratoma is observed (a, white arrow). This structure is particularly well-defined on sagittal (b, white arrow) and coronal (c, white arrow) images. A whirlpool sign is visible, particularly on the coronal image, accompanied by an elongated enhancement suspected to be the ovarian artery within it (twisted vascular pedicle) (c, green arrow). **d** is a laparoscopic image, which reveals a 540° torsion of the right mature cystic teratoma

Abnormal positioning of the adnexal structures is another key finding (Figs. 10–12, 15, and 16), which is reported in approximately 60% of cases [8, 12]. The affected ovary may be displaced to the midline, contralateral side, or even superior to its normal position [12, 26]. This displacement occurs because the ovary twists around its vascular pedicle, often pulling it away from its typical anatomical location [8, 12, 26]. Right-sided torsion is more common, possibly because of the presence of the sigmoid colon on the left side, which limits mobility [8, 12].

Uterine deviation toward the affected side was observed in 36%–70% of cases (Fig. 7i) [8, 12, 17, 18, 21, 27]. This finding is due to the traction effect of the twisted adnexa on the broad ligament, which causes the uterus to deviate toward the torsion side [8, 12, 18]. However, in some cases, particularly with large masses, the uterus may be displaced to the contralateral side [8, 12, 50].

Surrounding inflammatory changes manifest as haziness or stranding of the adjacent fat on CT and increased T2 signal intensity on MRI [12, 20, 21]. These alterations reflect the local inflammatory response to compromised blood flow and ischemia development [15, 21, 51, 52]. The severity of inflammatory alterations is often correlated with the duration of torsion [12, 21].

Free pelvic fluid is present in approximately 50% of cases [12, 50]. The amount of fluid can vary from trace amounts to significant accumulation, and its presence may reflect the severity and duration of vascular compromise [10, 12]. The fluid may show high attenuation on CT or high signal intensity on T1-weighted MRI in cases of hemorrhagic infarction [11, 20, 21].

#### CT-specific features of adnexal torsion

CT provides rapid acquisition and wider availability in emergency settings. It is particularly useful when the clinical presentation is ambiguous and other acute abdominal conditions should be excluded [12, 14, 26].

Hemorrhagic infarction is a critical finding indicating the nonviability of the affected adnexa. On unenhanced CT, areas of hemorrhage appear as high-attenuation foci (50–90 HU) within the enlarged ovary, as shown in Fig. 7h [11, 17, 21].

In cases of complete torsion, contrast-enhanced CT typically shows the absence or decreased enhancement of the affected ovary [12, 17, 20, 26]. A thin rim of enhancement may persist because of capsular perfusion, which is analogous to the cortical rim sign observed in renal infarction [4, 12].

The enhancement patterns vary with the degree and chronicity of torsion. Early torsion may exhibit preserved enhancement because of the dual blood supply from the ovarian and uterine arteries [10, 17, 25]. Progressive torsion leads to heterogeneous enhancement patterns, with areas of decreased enhancement reflecting compromised blood flow [14, 15, 17, 26].

The step-ladder pattern of the ovarian vessels represents the twisted vascular pedicle coursing between the uterus and affected adnexa (Fig. 12a) [26]. Coronal reformations improve the detection of the twisted pedicle, with reported sensitivity increasing from 28 to 78% [53].

Fat stranding in the surrounding pelvic tissue reflects inflammatory changes and is more prominent in patients with prolonged torsion (Fig. 7i). This finding is nonspecific, but it can help determine the chronicity of torsion [12, 21, 26, 54]. CT can effectively reveal vascular complications and associated findings, such as hemoperitoneum [4, 12, 15, 26].

#### MRI-specific features of adnexal torsion

MRI provides superior soft-tissue characterization of torsion-related changes. On T2-weighted images, ovarian stroma typically demonstrates increased signal intensity reflecting edema (Figs. 7b, 9a, and 9i), whereas T1-weighted images may show areas of high signal intensity indicating hemorrhage (Figs. 7e and 9d) [19, 20, 27, 55]. The evolution of hemorrhage follows the expected pattern of blood products on MRI, which helps determine the timeline of torsion [20, 56].

Susceptibility-weighted imaging is particularly useful for detecting hemorrhage and thrombosis within the twisted vascular pedicle [57]. Diffusion-weighted imaging (DWI) has emerged as a valuable tool for assessing ovarian viability. Restricted diffusion, manifested as high signal intensity on DWI with corresponding low apparent diffusion coefficient (ADC) values (Figs. 7c, 7 d, 9b, 9c, 9j, and 9k), suggests ischemic changes [13, 27, 58]. However, interpretation requires correlation with other sequences because hemorrhage can also cause restricted diffusion [15, 21, 55, 58].

Dynamic contrast-enhanced MRI can demonstrate various enhancement patterns. The complete absence of enhancement strongly suggests nonviability (Fig. 7f), whereas delayed or heterogeneous enhancement indicates partial torsion [17, 55]. The perifollicular T2 hypointense rim sign, representing hemorrhage within the theca layer of ovarian follicles (Fig. 7b), has been reported as a specific marker of hemorrhagic infarction and is correlated with reduced ovarian viability [17, 55, 56].

**Fig. 13:**
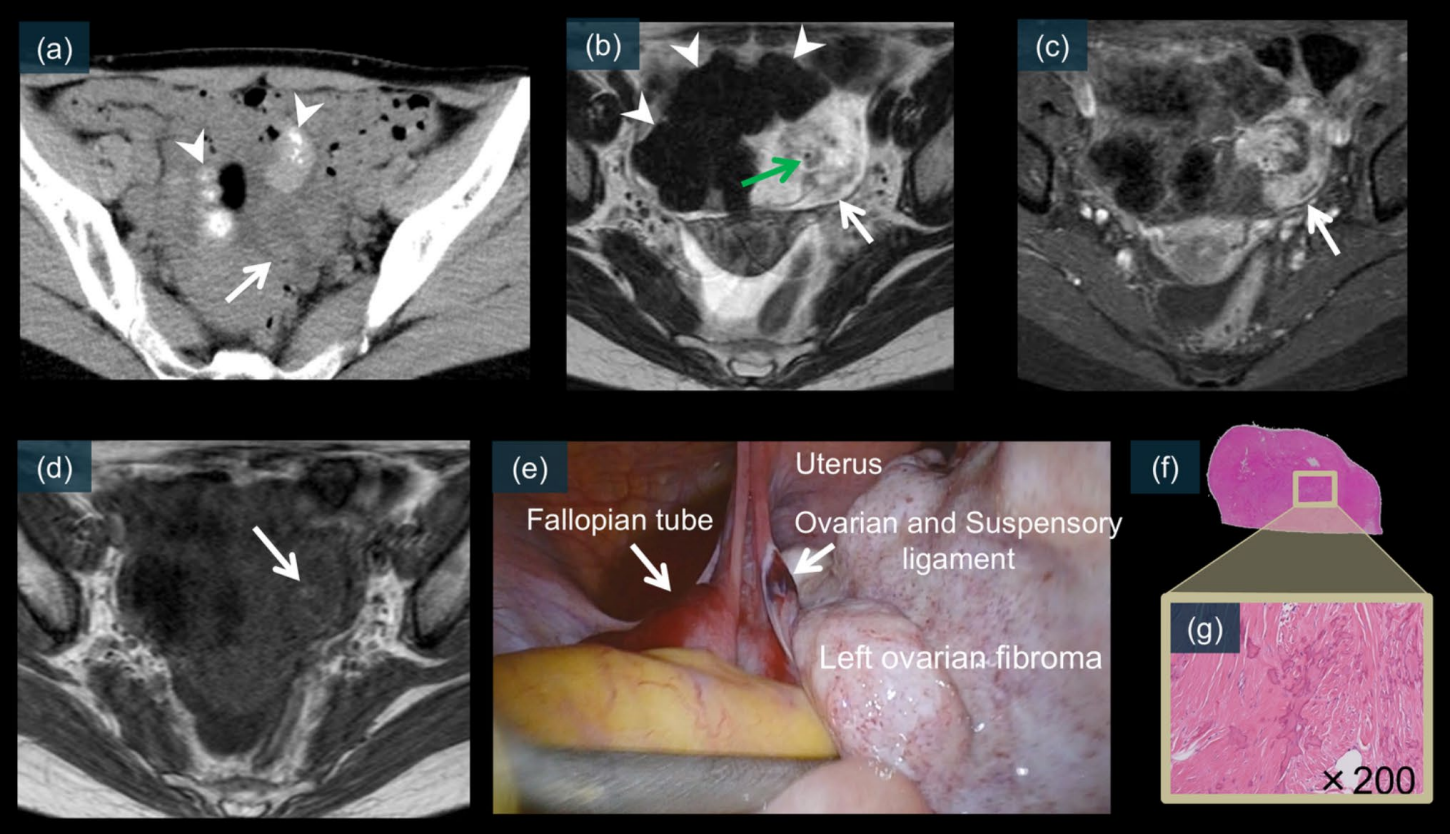
A 48-year-old female. **a** is an unenhanced CT image showing the superior portion of the ovarian fibroma with strong calcification (white arrowheads), adjacent to which a mass-like swelling is observed (white arrow). On T2WI (**b**), a thickened, edematous pedicle (white arrow) is observed adjacent to a low-signal mass suggestive of left ovarian fibroma (white arrowheads). A flow void suspected to be the ovarian artery is visible in the center of the pedicle (green arrow). This area shows a relatively strong enhancement (**c**, white arrow). On T1WI (**d**), a partially high-signal area was observed, suggesting hemorrhage (white arrow). **e** is a laparoscopic image revealing a 720° torsion of the left ovarian fibroma and fallopian tube. Pathological examination showed abundant thick collagen fibers with calcification deposits (f and g)

**Fig. 14:**
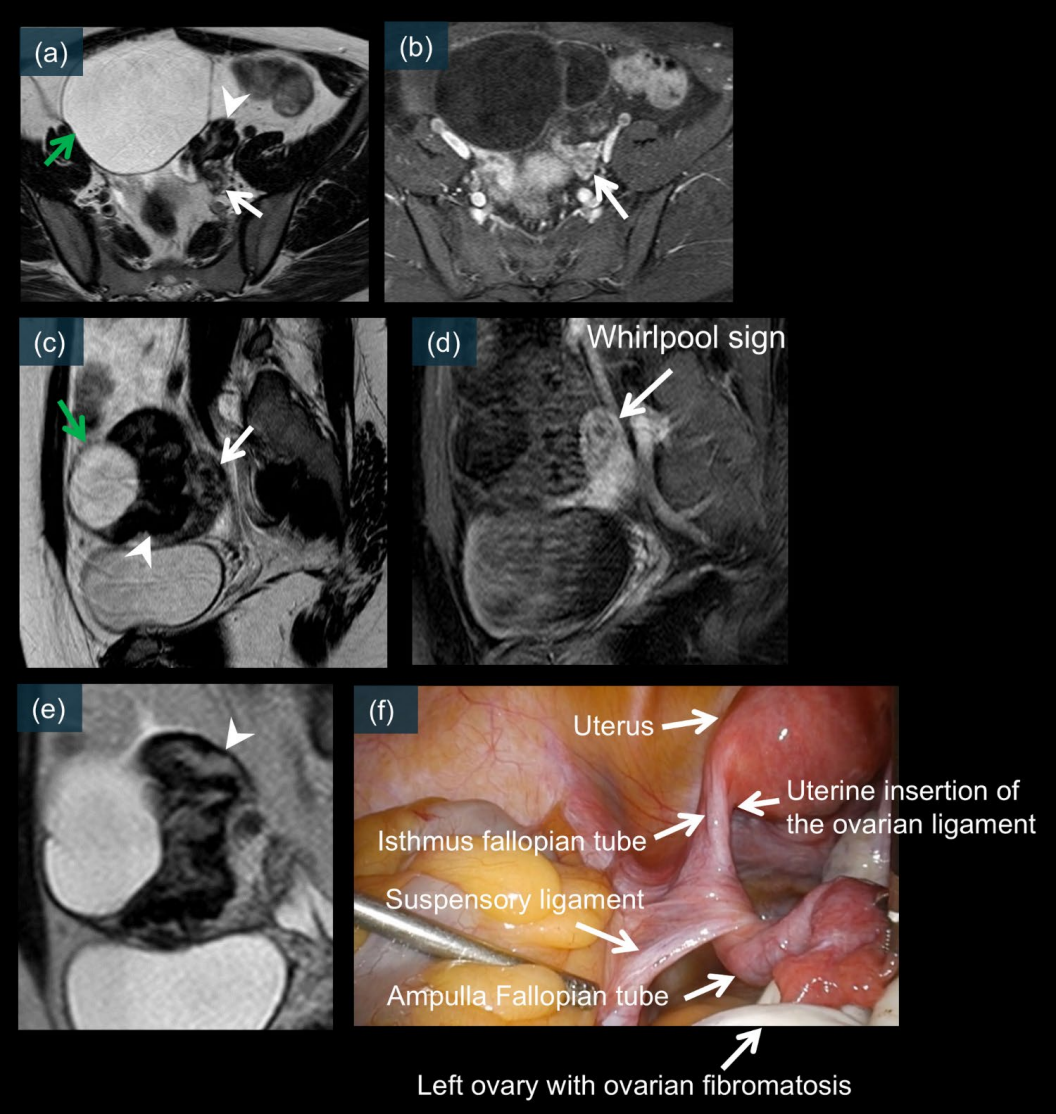
A 29-year-old female. **a–d**) show pelvic MR images. On T2WI (a and c), a multilocular cystic mass is observed in the left ovary (green arrows). Furthermore, a component with low signal intensity at the periphery on T2WI is noted (a, c and e, white arrowheads), which is considered to be the black garland sign. A twisted pedicle is also seen contiguous with this structure (a–c, white arrows). A whirlpool sign is clearly visible on the contrast-enhanced fat-suppressed T1WI sagittal image (d). **f** is a laparoscopic image, which reveals a 540° torsion of the left ovarian mucinous cystadenoma

The superior soft-tissue contrast of MRI allows better delineation of the twisted pedicle (Figs. 7b, 9f, g, m, n, 10e, 11f–k, 13b, c, and 14a–d) and its relationship to adjacent structures [8, 14, 26, 27]. Thickening of the fallopian tube and cystic mass walls is also better characterized on MRI because of the superior soft-tissue contrast (Fig. 7a) [14, 15]. This approach is particularly valuable in patients in whom CT findings are equivocal or when radiation exposure is a concern, such as pregnant patients [14, 15, 21, 52].

#### Essential diagnostic signs of adnexal torsion

##### Whirlpool sign

**Fig. 15:**
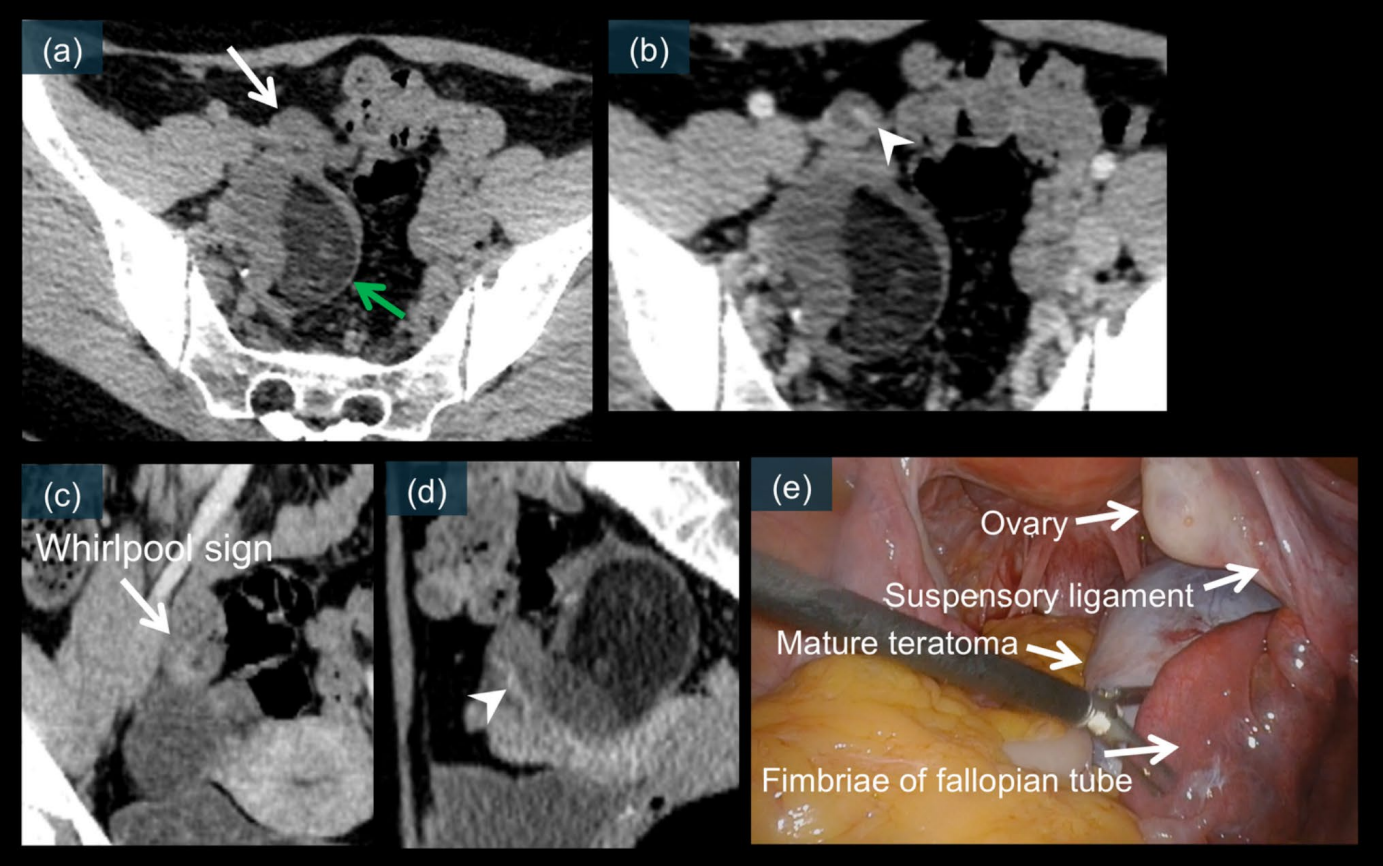
A 35-year-old female. **a** is an unenhanced CT image. **b–d** are contrast-enhanced CT images. The mature cystic teratoma is located in the midline of the pelvis. On unenhanced CT, a mass-like structure (white arrow) is observed adjacent to the mature cystic teratoma (green arrow) (a). On contrast-enhanced CT, particularly in the coronal plane, a whirlpool sign is clearly visualized (c, white arrow). The whirlpool sign is not clearly visible in the axial and sagittal planes (b and d). There is an elongated enhancement within the structure, suspected to be the ovarian artery (b and d, white arrowhead). **e** is a laparoscopic image, which reveals a 360° torsion of the mature cystic teratoma of the right fallopian tube. Furthermore, the ovarian ligament and ovary are not twisted

**Fig. 16:**
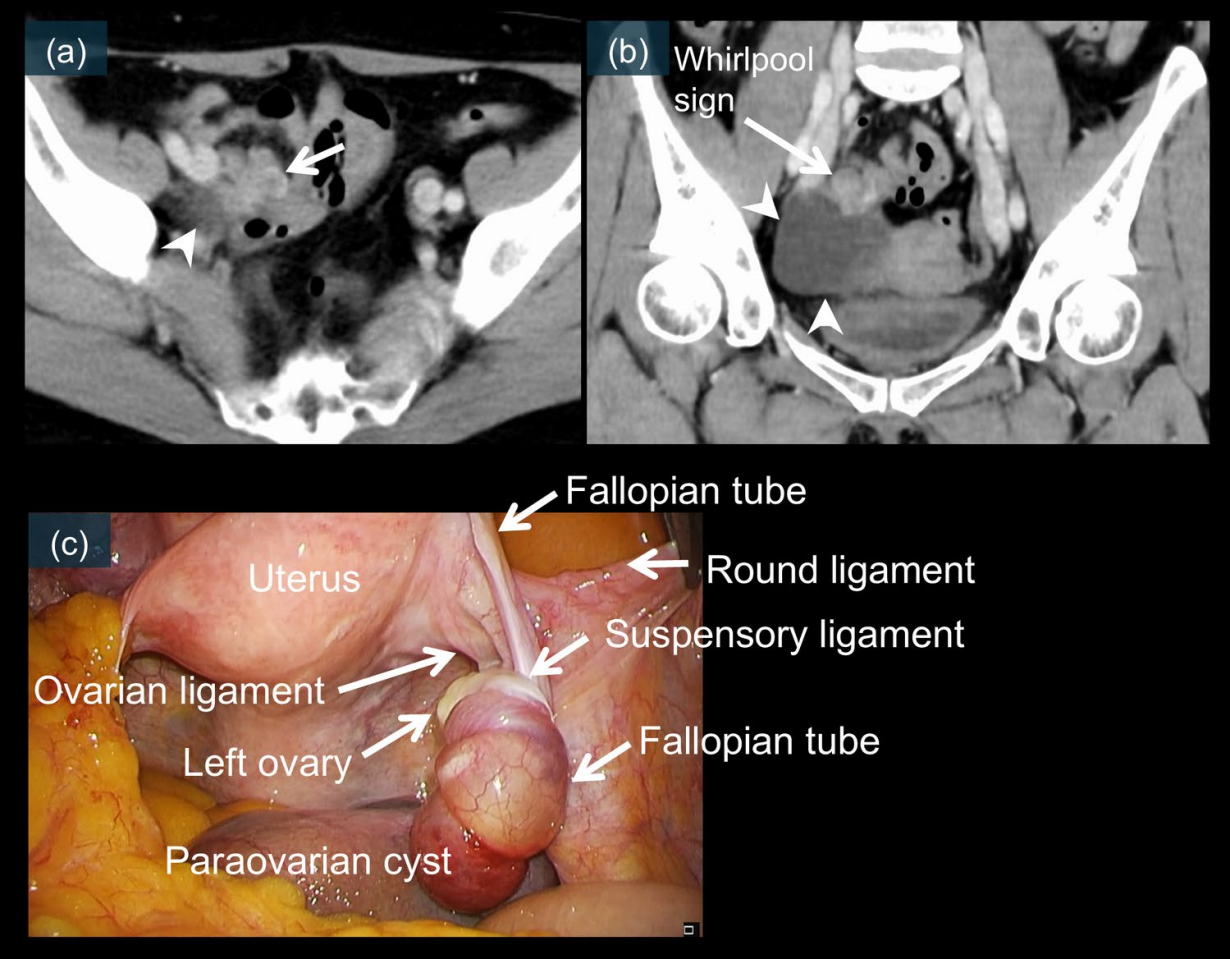
A 60-year-old female. Contrast-enhanced CT images (**a** and **b**) show a 4.4-cm mass in the pelvis (white arrowheads). Adjacent to the mass, a whirlpool sign is observed (b), which is considered to be a twisted pedicle (a and b, white arrows). (**c**) is a laparoscopic image, which reveals a 720° torsion of the right paraovarian cyst

The whirlpool sign represents the twisted pedicle and is considered a specific imaging finding of adnexal torsion (Figs. 7g, 9f, m, n, 10e, 12c, 14d, 15c, and 16b). On CT and MRI, the whirlpool sign appears as a spiral-shaped soft-tissue structure between the uterus and affected adnexa. On contrast-enhanced images, it typically demonstrates a thickened and enhanced pedicle with a twisted configuration [19–21, 24, 26, 28, 53]. The sensitivity of detecting this sign significantly increases with multiplanar imaging, particularly on coronal reformations, increasing from 28 to 78% [53]. Not all torsion cases demonstrate the classic whirlpool sign, despite the presence of a twisted pedicle. When visible, the whirlpool sign exhibits high specificity and a positive predictive value for torsion [8, 50]. Laparoscopic correlation shows a direct correspondence between the imaging appearance and the actual twisted pedicle (Figs. 9h, o, 10f, g, 11l, 12d, 14f, 15e, and 16c) [52].

##### String of pearls sign

This characteristic finding represents peripherally displaced ovarian follicles due to stromal edema, which is observed in up to 74% of adnexal torsion cases (Fig. 7b) [8, 10, 49]. On CT, it appears as multiple peripheral cystic structures within an enlarged ovary [8, 12]. MRI shows this sign more clearly, particularly on T2-weighted images in which the follicles appear as high-signal-intensity structures against the background of edematous stroma [8, 27, 55, 56]. The presence of this sign is nonspecific for torsion; however, it helps distinguish ovarian from nonovarian masses and indicates significant ovarian congestion [10, 49].

##### Additional important signs of adnexal torsion

The beak sign refers to the tapering of the twisted pedicle as it connects to the affected adnexa, creating a characteristic pointed appearance [8, 12, 53]. This finding is particularly well demonstrated on cross-sectional imaging and helps identify the point of torsion [53].

Adnexal cyst wall thickening patterns can help differentiate the underlying pathology: smooth and concentric wall thickening suggests simple edema, particularly when associated with peripheral cystic structures, whereas eccentric or irregular wall thickening exceeding 10 mm indicates hemorrhagic infarction [20, 21, 55].

Fallopian tube thickening exceeding 10 mm is a significant finding indicating vascular congestion and edema (Fig. 7a) [12, 14, 20].

### Leiomyoma torsion

#### CT and MRI features of leiomyoma torsion

**Fig. 17:**
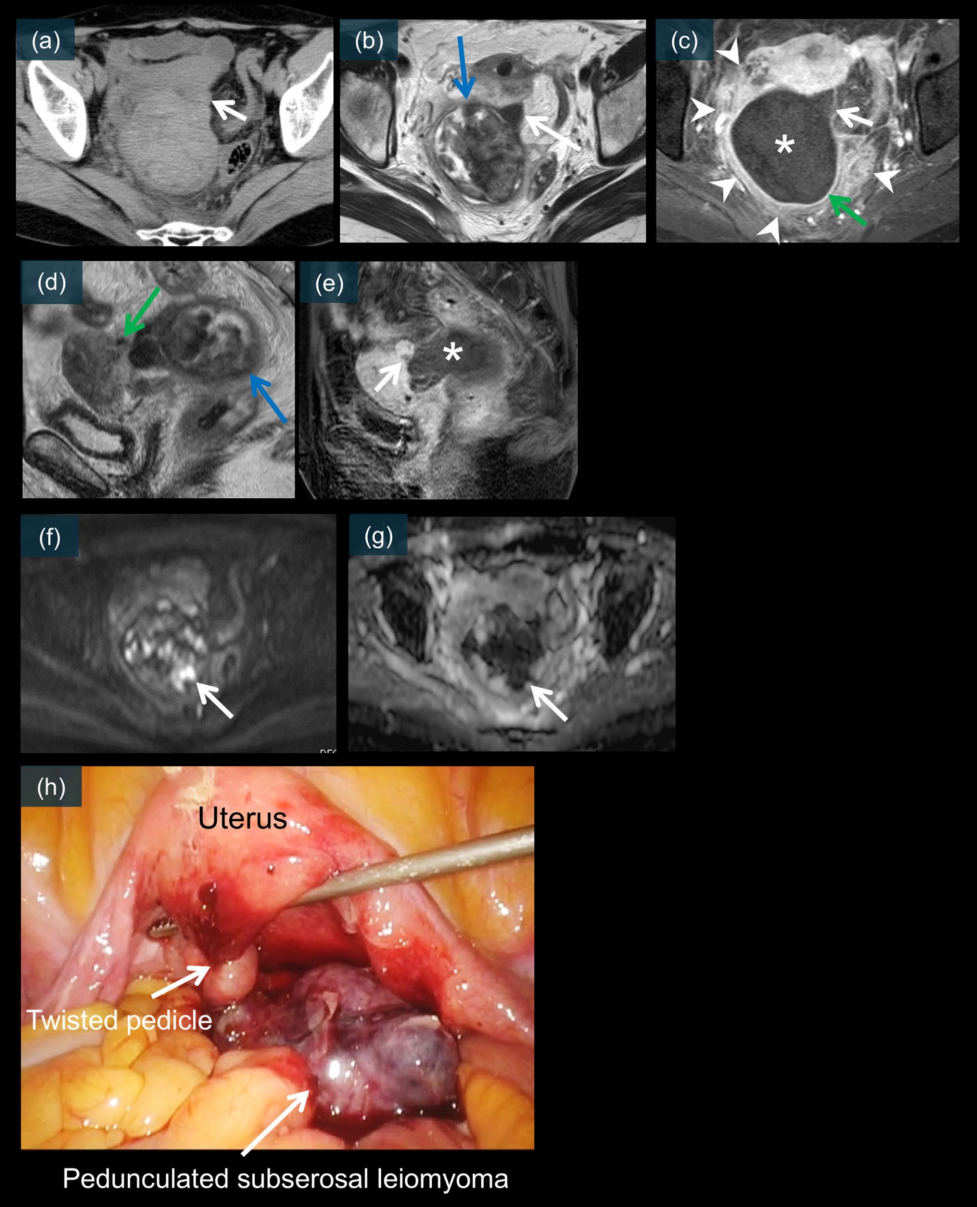
A 66-year-old female. An unenhanced CT scan (**a**) shows a mass with a long diameter of 7.4 cm in the pelvic cavity, which appears to be partially continuous with the uterus (white arrow). On T2WI (**b** and **d**), the structure continuous with the uterus is clearly depicted and can be recognized as an enlarged stalk in the axial view (white arrow). In the sagittal view, a bridging vessel sign is observed (green arrow). The mass shows a heterogeneous internal signal intensity (blue arrows). On the contrast-enhanced images (**c** and **e**), neither the stalk nor the mass showed enhancement (white arrows and asterisks). A thin enhanced rim is observed at the periphery (green arrow). The nonenhanced area corresponds to necrosis, and the usual rim of enhancement corresponds to obstructed peripheral veins with edema. There is also enhancement around this area, suggesting inflammation (white arrowheads). DWI/ADC (**f** and **g**) show partial diffusion restriction within the mass, corresponding to ischemia and hemorrhagic necrosis (white arrows). (**h**) is a laparoscopic image showing a subserosal leiomyoma that has undergone a 720° torsion

Leiomyoma torsion presents with distinctive imaging features that differ from those of adnexal torsion. On unenhanced CT, a pedunculated subserosal leiomyoma is typically visualized as a well-defined mass connected to the uterus via a stalk (Fig. 17a). The mass may exhibit heterogeneous density, reflecting degenerative changes [3, 4].

**Fig. 18:**
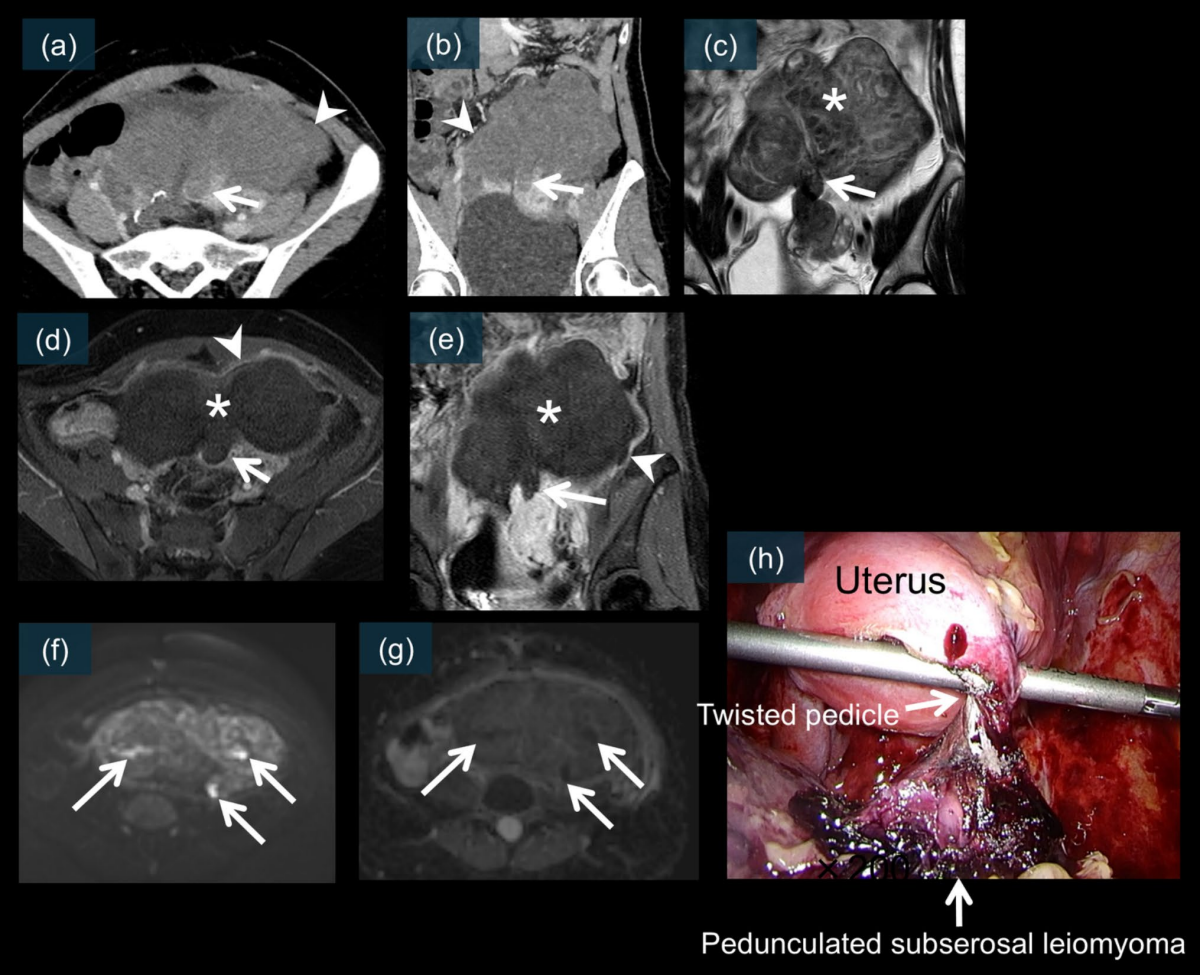
Contrast-enhanced CT images of a 35-year-old patient. CT images (**a** and **b**) show a 1.5 cm pelvic mass connected to the uterus by a stalk. The mass demonstrates poor internal enhancement with a thin peripheral rim of enhancement (white arrowheads). A low contrast enhancement area is observed in the uterine region adjacent to the mass, which is called the “dark fan sign (white arrows).” This CT finding is believed to reflect the decreased perfusion of the uterine muscle adjacent to the subserosal leiomyoma with acute torsion. On T2WI (**c**), the stalk connecting to the uterus is clearly depicted and appears slightly enlarged (white arrow). The mass shows a heterogeneous internal signal intensity (white asterisk). On contrast-enhanced MRI (**d** and **e**), neither the non-enhancing wedge-shaped area (white arrows) corresponding to the “dark fan” sign nor the mass itself shows enhancement (white asterisks). A thin enhanced rim is observed at the periphery (white arrowheads). The nonenhanced area corresponds to necrosis, and the usual rim of enhancement corresponds to obstructed peripheral veins with edema. DWI/ADC (**f** and **g**) shows partial diffusion restriction within the mass, corresponding to ischemia and hemorrhagic necrosis (white arrows). (**h**) is a laparoscopic image showing a subserosal leiomyoma that has undergone a 480° torsion

On contrast-enhanced CT, the twisted leiomyoma typically demonstrates poor internal enhancement with thin peripheral rim enhancement (Figs. 8a, 18a, and b) [48]. This pattern indicates compromised blood supply to the central portion of the leiomyoma while preserving some capsular perfusion [3, 4]. The stalk connecting the leiomyoma to the uterus may appear thickened and edematous with variable enhancement (Figs. 8a and 18a–b) [4, 48].

On MRI, leiomyoma torsion exhibits characteristic signal abnormalities. T2-weighted images typically show heterogeneous internal signal intensity (Figs. 8c, d, 17b, d, and 18c), with areas of increased signal reflecting edema and degeneration [3, 59, 60]. The stalk is usually well visualized on both T2-weighted and contrast-enhanced images (Figs. 8c–e, 17b–e, and 18c–e). It appears thickened and shows the “bridging vessel sign” (Figs. 8c and 17d) [3, 60].

Postcontrast MRI typically shows absent or decreased enhancement within the leiomyoma with thin rim enhancement (Figs. 8b, e, 17c, 18d, and e) [3, 59]. This pattern is similar to that observed on CT and indicates the degree of vascular compromise [3, 4].

DWI often shows restricted diffusion within the leiomyoma (Figs. 8f, g, 17f, g, 18f, and g), indicating ischemic changes [3, 59, 60]. This finding, when combined with the enhancement pattern, can help assess the degree of ischemia and potential viability of the leiomyoma tissue.

#### Essential diagnostic signs of leiomyoma torsion

##### Dark fan sign

The dark fan sign is a relatively recently described finding specific to leiomyoma torsion. This sign represents decreased enhancement of the uterine myometrium adjacent to the twisted pedunculated leiomyoma (Figs. 8a, 18a, and b) [48]. This sign is best appreciated on contrast-enhanced CT images and reflects compromised perfusion in the affected myometrium [48]. The presence of this sign demonstrates high specificity (100%) for leiomyoma torsion and is well correlated with surgical findings [48]. The pathophysiological basis involves the mechanical compression of myometrial vessels by the twisted pedicle [4].

##### Bridging vessel sign

The bridging vessel sign is best visualized on T2-weighted MRI and indicates the twisted vascular pedicle connecting the leiomyoma to the uterus (Figs. 8c and 17d) [3, 60]. This sign provides important information about the point of torsion and the vascular supply to the leiomyoma [3].

Laparoscopic examination revealed the pedunculated leiomyoma with its twisted stalk, often showing color changes indicating the degree of ischemia (Figs. 17h and 18h) [5, 39]. The degree of torsion and duration of symptoms are correlated with the extent of tissue alterations observed during surgery [3, 5].

## Comparative analysis of imaging modalities

Table 1 presents a detailed comparison of the CT and MR imaging features of adnexal and leiomyoma torsion.

**Table 1:** Comparison of CT and MR Imaging Features in Adnexal and Leiomyoma Torsion

Adnexal Torsion Features	MRI	CT	Notes
Ovarian enlargement	✓	✓	
Twisted pedicle (Whirlpool sign)	✓	✓	Often more visible on MRI
Fallopian tube thickening	✓	✓	
Uterine deviation	✓	✓	
Smooth wall thickening of cystic mass	✓	✓	
Pelvic free fluid	✓	✓	
Abnormal ovarian position	✓	✓	
Peripherally displaced follicles (string of pearls)	✓	✓	More clearly visible on MRI
Lack of enhancement	✓	✓	MRI more sensitive for subtle changes
Stromal edema	✓	△	Better characterized on MRI (T2 signal)
Hemorrhage within ovary	✓	△	Better characterized on MRI (T1 signal)
Hematosalpinx	✓	△	Better characterized on MRI
Perifollicular T2 hypointense rim	✓	✗	MRI-specific finding
Diffusion restriction on DWI	✓	✗	MRI-specific finding
Susceptibility artifacts on SWI	✓	✗	MRI-specific finding
Leiomyoma Torsion Features			
Poor internal enhancement	✓	✓	
Thin rim enhancement	✓	✓	
Thickened stalk	✓	✓	
Dark fan sign	✓	✓	Traditionally described on CT; equivalent finding visible on MRI
Bridging vessel sign	✓	△	Primarily an MRI term; vascular structures may be visible on CT but the term is not commonly used in CT interpretation
Diffusion restriction on DWI	✓	✗	MRI-specific finding
GENERAL MODALITY CHARACTERISTICS			
Detailed soft-tissue characterization	✓	✗	MRI superior
Radiation exposure	✗	✓	CT involves ionizing radiation
Speed of acquisition	✗	✓	CT faster than MRI
Availability in emergency settings	✗	✓	CT more widely available

CT, computed tomography; DWI, diffusion-weighted imaging; MRI, magnetic resonance imaging; SWI, susceptibility-weighted imaging

✓: clearly visible/applicable

△: may be visible but less clear/sensitive

✗: not visible/applicable

CT and MRI offer distinct advantages in the diagnosis of adnexal and leiomyoma torsion. Although both modalities can demonstrate key features such as ovarian enlargement and the whirlpool sign, their specific strength warrants consideration in different clinical scenarios [17, 20].

MRI demonstrates superior soft-tissue contrast, allowing better characterization of the ovarian stroma and hemorrhage. The multiplanar capabilities and lack of ionizing radiation make it particularly valuable for young patients and pregnant women [14, 18]. MRI excels at detecting subtle changes, such as stromal edema on T2-weighted images and early hemorrhagic changes on T1-weighted sequences. Susceptibility-weighted imaging is particularly useful for detecting hemorrhage and thrombosis within the twisted vascular pedicle [57]. Furthermore, the perifollicular T2 hypointense rim sign, which may predict ovarian viability, is best appreciated on MRI [15, 56].

The use of DWI adds significant diagnostic value because infarction areas typically demonstrate restricted diffusion. This finding, combined with contrast enhancement patterns, can help assess tissue viability [13, 55, 58]. Fallopian tube thickening and wall thickening of cystic masses are also better characterized on MRI because of its superior soft-tissue contrast [14, 15].

CT involves ionizing radiation; however, it offers rapid acquisition and wider availability in emergency settings. It is particularly useful when the clinical presentation is ambiguous and other acute abdominal conditions should be excluded [12, 14, 26]. The dark fan sign in leiomyoma torsion is best appreciated on CT, and coronal reformations have been shown to improve the detection of the twisted pedicle, with the reported sensitivity increasing from 28 to 78% [48]. Furthermore, CT can effectively demonstrate vascular complications and associated findings, such as hemoperitoneum [4, 12, 15, 26].

The choice between these modalities often depends on clinical factors, including patient age, pregnancy status, clinical stability, and the need for emergency intervention. Although both modalities can effectively diagnose torsion, MRI provides more detailed tissue characterization when time permits, whereas CT offers rapid assessment in acute settings.

## Specific types of torsion with image–surgery correlation

### Ovarian torsion variants

#### Massive ovarian edema

Massive ovarian edema is a rare entity characterized by a partially torsed ovary with intermittent vascular compromise. It typically occurs in young women and can be mistaken for an ovarian neoplasm [15, 55, 61, 62]. The imaging features included marked enlargement of the ovary with preservation of the follicular structure at the periphery and T2 hyperintense stromal edema on MRI (Figs. 9a and i). DWI shows areas of restricted diffusion, indicating edema and potential hemorrhagic changes (Figs. 9b, c, j, and k) [13, 55, 56, 58]. In postcontrast sequences, enhancement is typically observed in the periphery of the ovaries and follicles (Fig. 9e and l) [15, 55]. Laparoscopic examination revealed an enlarged edematous ovary with multiple peripheral follicles (Fig. 9h and o) [62]. Early recognition is crucial because this condition can often be managed conservatively with detorsion [15, 63]. Figure 9 presents the characteristic imaging and laparoscopic findings of massive ovarian edema.

### Ovarian mature cystic teratoma

Mature cystic teratoma is the most common ovarian neoplasm to undergo torsion [10, 20, 41]. The fatty component of these tumors may predispose patients to torsion by causing the ovary to “float” within the pelvis [8]. CT can effectively demonstrate the characteristic whirlpool sign, indicating a twisted vascular pedicle (Figs. 11e and 12c) [24, 26, 53]. MRI provides additional diagnostic value by revealing T2 signal changes of the twisted pedicle (Figs. 10e and 11f–k) [19, 21]. Contrast-enhanced imaging may reveal a lack of enhancement within the twisted vascular pedicle or surrounding ovarian tissue (Fig. 12b) [17, 21]. Laparoscopic examination typically reveals a twisted, enlarged ovary containing the teratoma, often with multiple rotations of the vascular pedicle. Early recognition is particularly important because affected patients may be suitable for organ-preserving surgery if diagnosed promptly (Figs. 10f, g, 11l, and 12d) [31, 41]. Figures 10–12 present the characteristic CT whirlpool sign, MRI signal changes of the twisted pedicle, and laparoscopic findings of a mature torsed ovarian teratoma, respectively.

### Ovarian fibroma

Ovarian fibroma is an uncommon sex cord-stromal tumor that can undergo torsion [51]. Although these solid tumors are less commonly associated with torsion than cystic lesions, their dense nature and weight can predispose them to this complication [17]. On unenhanced CT, fibromas typically appear as well-circumscribed solid masses that may show a mass-like swelling when torsed (Fig. 13a) [18, 64]. MRI demonstrates characteristic low signal intensity on T2-weighted images, reflecting their fibrous nature, with a thickened edematous pedicle in cases of torsion (Fig. 13b) [51, 65]. Contrast-enhanced imaging may only show the peripheral enhancement of the twisted pedicle (Fig. 13c) [17, 66]. Laparoscopic examination revealed a solid ovarian mass with a characteristic white–gray appearance and multiple rotations of the vascular pedicle (Fig. 13e) [65, 67]. Figure 13 presents a well-circumscribed solid ovarian mass with characteristic low T2 signal intensity and a twisted pedicle on MRI, which are typical of a torsed ovarian fibroma.

### Ovarian mucinous cystadenoma

Mucinous cystadenoma can present with torsion because of its often large size and cystic nature [17, 20]. Cross-sectional imaging is particularly valuable in these cases because the mass size may limit ultrasound evaluation [14]. MRI demonstrates characteristic findings, including a T2-weighted hyperintense multilocular cystic mass (Fig. 14a and c) with a twisted pedicle and variable enhancement patterns after contrast administration (Fig. 14b and d) [18, 55]. Laparoscopic examination typically reveals an enlarged, multicystic mass with torsion of the vascular pedicle, and the presence of ovarian fibromatosis suggests previous episodes of intermittent torsion (Fig. 14f) [10, 19, 20]. Figure 14 shows a large, multilocular cystic mass with a characteristic twisted pedicle on MRI, representing a torsed ovarian mucinous cystadenoma.

### IFTT

IFTT is a rare condition with unique imaging and surgical characteristics. As previously described in the “Mechanisms” section, IFTT can be classified into three subtypes based on the axis and presence of a leading mass (Fig. 4) [15, 30]: Type 1, organoaxial form without a leading mass; Type 2, organoaxial form with a leading mass; and Type 3, mesenteroaxial form. In our experience, Type 2 IFTT is the most commonly encountered form and is typically associated with paratubal cysts or, rarely, fallopian tube tumors [15, 30].

Two Type 2 IFTT variants are presented in this review. The first variant is Type 2 IFTT with fallopian tube teratoma, which is an extremely rare entity. This variant presents with mature cystic teratoma characteristics with normal ipsilateral ovary (Fig. 15). A whirlpool sign is clearly visualized on coronal CT images (Fig. 15c), and laparoscopic examination reveals a twisted fallopian tube containing a teratoma with a normal ovary (Fig. 15e) [15, 30, 68].

The second variant is Type 2 IFTT with a paraovarian cyst. Key imaging features include a midline cystic mass with an adjacent twisted pedicle (Fig. 16a and b) and a normal ipsilateral ovary separate from the torsed structure. Laparoscopic examination confirms a torsed paraovarian cyst with a normal adjacent ovary (Fig. 16c) [15, 30, 38, 69].

### Leiomyoma torsion

Torsion of a pedunculated subserosal leiomyoma is a rare but significant complication that requires prompt recognition and surgical intervention [3, 4, 59]. CT demonstrates several key features, including poor contrast enhancement inside the leiomyoma, thin rim enhancement around the mass, and a characteristic “dark fan sign” representing decreased perfusion in the adjacent myometrium (Fig. 18a and b) [48]. MRI findings include a nonenhanced stalk with a thin enhanced rim (Figs. 17c, e, 18d, and e), and diffusion restriction may be present, reflecting ischemic changes [3, 4, 59, 60]. The “bridging vessel sign” on T2-weighted images represents the twisted vascular pedicle connecting the leiomyoma to the uterus (Fig. 17d) [3, 60]. Laparoscopic examination revealed a pedunculated leiomyoma with variable degrees of torsion and color changes reflecting the extent of ischemia [5, 39]. Figures 17 and 18 show cases of pedunculated subserosal leiomyoma torsion, each showing cross-sectional imaging findings including the “dark fan sign” with poor internal enhancement and thin rim enhancement, along with corresponding laparoscopic images revealing the twisted, ischemic leiomyomas.

## Conclusion

Understanding the correlation between imaging findings and laparoscopic appearance is crucial for accurate diagnosis of adnexal and leiomyoma torsion. Key imaging features, such as the whirlpool sign, dark fan sign, and specific enhancement patterns, can be integrated with anatomical knowledge to enable prompt and accurate diagnosis. Different types of torsion present unique imaging and surgical characteristics, including massive ovarian edema, various ovarian tumors with torsion, IFTT, and leiomyoma torsion. The recognition of these specific patterns and their laparoscopic correlates can significantly improve diagnostic accuracy and guide appropriate surgical management, ultimately leading to better patient outcomes.
